# Tracking the Dairy Microbiota from Farm Bulk Tank to Skimmed Milk Powder

**DOI:** 10.1128/mSystems.00226-20

**Published:** 2020-04-07

**Authors:** Aoife J. McHugh, Conor Feehily, Mark A. Fenelon, David Gleeson, Colin Hill, Paul D. Cotter

**Affiliations:** a Food Bioscience Department, Teagasc Food Research Centre, Cork, Ireland; b School of Microbiology, University College Cork, Cork, Ireland; c APC Microbiome Ireland, Cork, Ireland; d Food Chemistry and Technology Department, Teagasc Food Research Centre, Cork, Ireland; e Teagasc Animal and Grassland Research and Innovation Centre, Cork, Ireland; University of California, San Diego

**Keywords:** dairy, processing, metagenomics, skimmed milk powder, 16S rRNA gene amplicon sequencing, bulk tank milk, whole-milk silo, skimmed milk silo, collection tanker, microbiota

## Abstract

Microorganisms from the environment can enter the dairy supply chain at multiple stages, including production, milk collection, and processing, with potential implications for quality and safety. The ability to track these microorganisms can be greatly enhanced by the use of high-throughput DNA sequencing (HTS). Here HTS, both 16S rRNA gene amplicon and shotgun metagenomic sequencing were applied to investigate the microbiomes of fresh mid- and late-lactation milk collected from farm bulk tanks, collection tankers, milk silos, skimmed milk silos, a cream silo, and powder samples to investigate the microbial changes throughout a skim milk powder manufacturing process. 16S rRNA gene analysis established that the microbiota of raw milks from farm bulk tanks and in collection tankers were very diverse but that psychrotrophic genera associated with spoilage, *Pseudomonas* and *Acinetobacter*, were present in all samples. Upon storage within the whole-milk silo at the processing facility, the species Pseudomonas fluorescens and Acinetobacter baumannii became dominant. The skimmed milk powder generated during the mid-lactation period had a microbial composition that was very different from that of raw milk; specifically, two thermophilic genera, *Thermus* and *Geobacillus*, were enriched. In contrast, the microbiota of skimmed milk powder generated from late-lactation milk more closely resembled that of the raw milk and was dominated by spoilage-associated psychrotrophic bacteria. This study demonstrates that the dairy microbiota can differ significantly across different sampling days. More specifically, HTS can be used to trace microbial species from raw milks through processing to final powdered products.

**IMPORTANCE** Microorganisms can enter and persist in dairy at several stages of the processing chain. Detection of microorganisms within dairy food processing is currently a time-consuming and often inaccurate process. This study provides evidence that high-throughput sequencing can be used as an effective tool to accurately identify microorganisms along the processing chain. In addition, it demonstrates that the populations of microbes change from raw milk to the end product. Routine implementation of high-throughput sequencing would elucidate the factors that influence population dynamics. This will enable a manufacturer to adopt control measures specific to each stage of processing and respond in an effective manner, which would ultimately lead to increased food safety and quality.

## Abstract (precis)

Microorganisms can enter and persist in dairy at several stages of the processing chain. Detection of microorganisms within dairy food processing is currently a time-consuming and often inaccurate process. This study provides evidence that high-throughput sequencing can be used as an effective tool to accurately identify microorganisms along the processing chain. In addition, it demonstrates that the populations of microbes change from raw milk to the end product. Routine implementation of high-throughput sequencing would elucidate the factors that influence population dynamics. This will enable a manufacturer to adopt control measures specific to each stage of processing and respond in an effective manner, which would ultimately lead to increased food safety and quality.

## INTRODUCTION

Bovine milk is a nutritious natural food that can be processed into many different products, including dairy powders that can be used as a base for therapeutic, nutritional, and/or infant formulas. Processing is required to provide a safe and stable shelf life and has a considerable impact on the microbial communities of the resultant products (1). With increased global demand for dairy products, including milk powders that are incorporated into infant milk formula, there is an even greater need to understand the associated microbiota in order to optimize food safety and quality. Such an understanding should incorporate an appreciation of the impact of both raw ingredients and processing environments on the final product (2–7). However, more information is needed to build a comprehensive view of the dairy microbiota and the factors that contribute to its composition. Traditional microbiological detection techniques focus on culturable bacteria. However, these approaches will not capture viable but nonculturable bacteria (1, 8) or non-readily-culturable bacteria (9). They can also be susceptible to false positives/negatives (10, 11), may not differentiate between closely related species, rely on a specific test for each target microbe, and are often time-consuming. More recently, high-throughput DNA sequencing (HTS) has been used to study the influence of environmental factors on the dairy microbiota. Animal housing, cleaning, and milking practices (7); weather conditions (12); seasonal influences and on-farm storage conditions (13); as well as large-scale storage and seasonal transportation (14) have been shown to influence the raw-milk microbiota. Seasonality can be particularly important in pasture-based systems when milk quality is impacted by where the herd is located (indoor versus outdoor), among other factors. In addition, associations have been made between the raw-milk microbiota and somatic cell counts (a hygiene and herd health indicator) in bulk tank (BT) samples (12, 15). The impacts of processing, such as pasteurization, on the milk microbiota (1) have been studied. However, more studies are needed to provide a complete view of different types of processing as well as the impact of different processing runs and different processing days on the dairy microbiota. Previous studies are limited by the use of 16S rRNA gene amplicon sequencing, which can provide taxonomic resolution to the genus level only. Therefore, there is an insufficient understanding of the functional potential of the microbial communities and, indeed, characterization of nonbacterial microbial contamination along the dairy chain. Recently, shotgun metagenomic sequencing, which overcomes these issues, has been used to study dairy products (16–18). Here, 16S rRNA gene amplicon and shotgun metagenomic analyses are used together to facilitate an in-depth study of the dairy microbiome from the farm through transportation and processing to a skimmed milk powder (SMP) and, in the process, provide valuable information regarding the impacts of collection, storage, and processing on this.

## RESULTS

### Bulk tank milks contain a diverse microbiota that differs in samples collected from the mid- and late-lactation periods.

**FIG 1:**
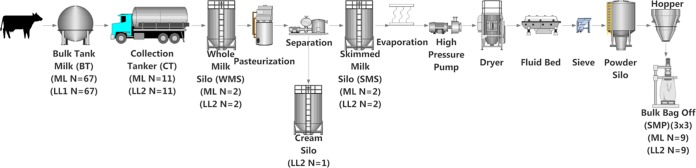
Sampling schematic. Sampling included mid-lactation (ML) samples (May 2016), where a whole processing run was sampled from farm bulk tank milk to skimmed milk powder. This included 67 farm bulk tanks, the 11 collection tankers used to collect this milk, and the whole-milk silo into which this milk was pooled (2 samples were obtained). Milk in the WMS was subjected to pasteurization and separation and stored in a skimmed milk silo from which 2 samples were obtained. Milk in the SMS was subjected to heating and drying to make a skimmed milk powder (SMP), from which 9 samples were obtained, 3 samples from each of 3 bags. The same 67 farms were resampled during late lactation (October 2016) (LL1, late lactation 1). On a separate day during this late-lactation period (December 2016) (LL2, late lactation 2), samples from tankers and the processing run, including an additional cream sample, were collected for analysis.

**FIG 2:**
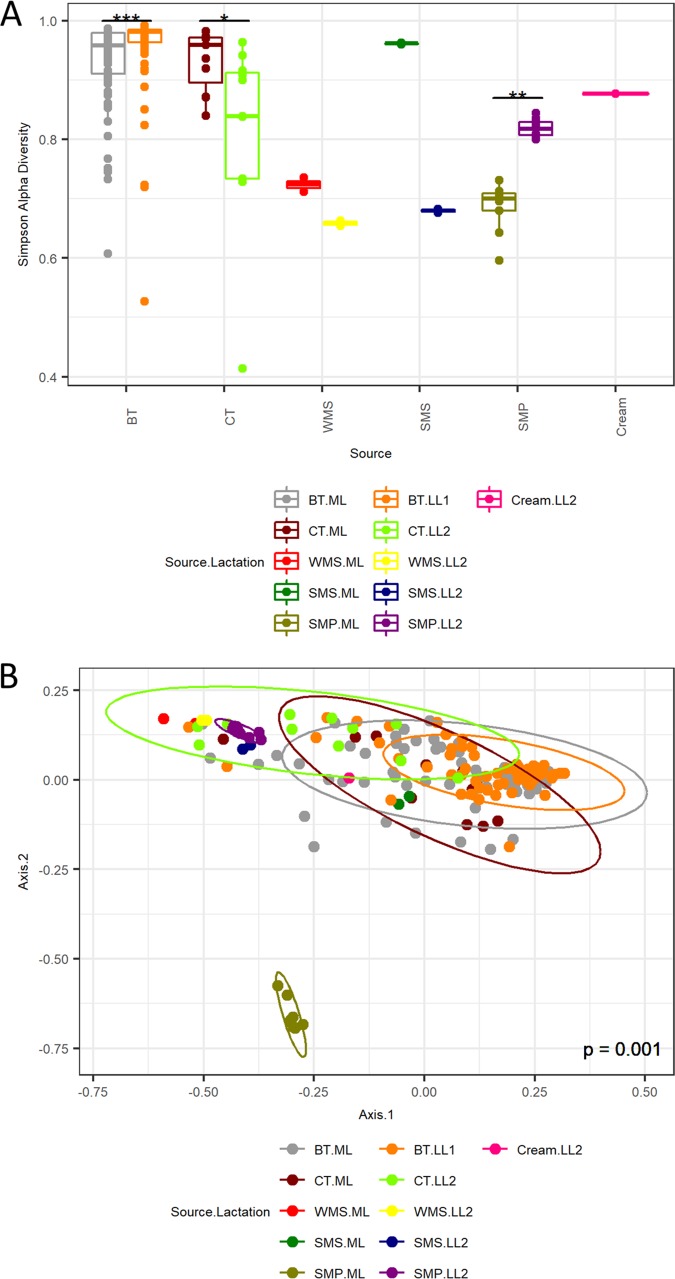
Microbial diversity indexes of sampling sites. (A) Simpson alpha diversity analysis of 16S rRNA gene amplicon sequence data for mid-lactation (ML) and late-lactation (LL1/LL2) samples from bulk tanks (BT), collection tankers (CT), whole-milk silos (WMS), skimmed milk silos (SMS), skimmed milk powder (SMP), and cream (LL2 only). Significant differences are highlighted (***, *P < *0.001; **, *P < *0.01; *, *P < *0.05). (B) Bray-Curtis multidimensional scaling analysis of 16S rRNA gene amplicon sequence data for mid- and late-lactation samples from bulk tanks, collection tankers, whole-milk silos, skimmed milk silos, skimmed milk powder, and cream (late lactation only).

**FIG 3:**
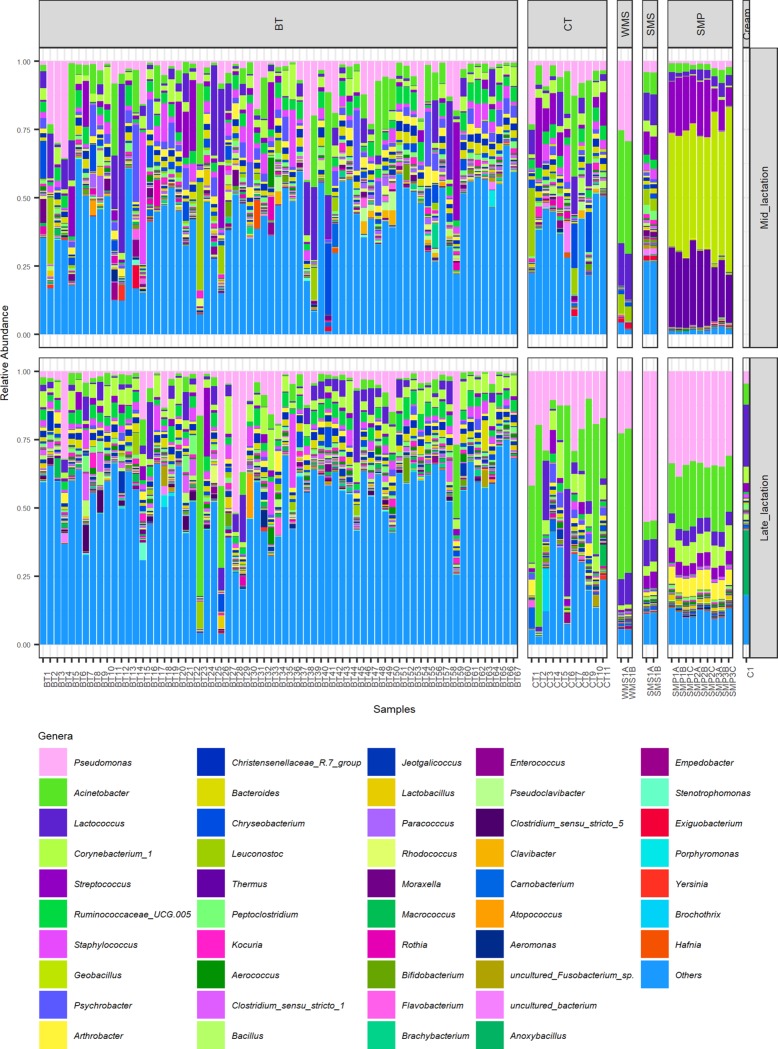
Genera present at a >5% relative abundance in at least one sample. Shown are 16S rRNA gene amplicon sequence data for mid- and late-lactation samples from bulk tanks (BT), collection tankers (CT), whole-milk silos (WMS), skimmed milk silos (SMS), skimmed milk powders (SMP), and cream (late lactation only). Genera shown are present at a >5% relative abundance in at least one sample.

**FIG 4:**
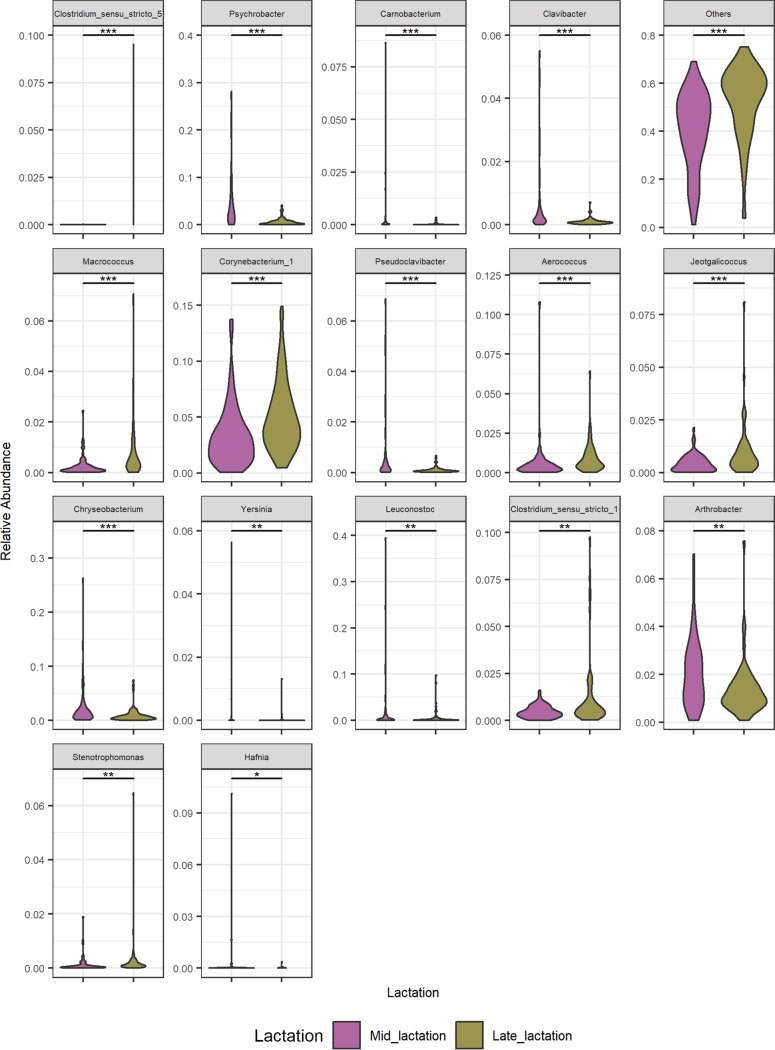
Significantly differential relative abundances of taxa constituting at least a 5% relative abundance in at least one sample between mid- and late-lactation bulk tanks. Shown are violin plots of genera that differ significantly in relative abundances between mid- and late-lactation bulk tanks. This highlighted genera that are present at a >5% relative abundance in at least one sample. A pairwise Wilcoxon rank sum test using Benjamini-Hochberg *P* value correction/FDR correction analysis was performed, and samples are ordered by the lowest *P* value to the highest (***, *P < *0.001; **, *P < *0.01; *, *P < *0.05).

DNA was extracted and 16S rRNA gene amplicons were sequenced for a total of 67 raw bulk tank (BT) milk samples collected on 1 day in the early- to mid-lactation period (May 2016), here referred to as mid-lactation (ML). This process was repeated in the late-lactation period (October 2016) (Fig. 1). The alpha diversity of the raw-milk microbiota in bulk tanks on farms was relatively high (Fig. 2A) compared to that in subsequent processing stages. However, the microbial alpha diversity in mid-lactation bulk tank samples was significantly lower (*P < *0.001) than that in the corresponding late-lactation samples (Fig. 2A). Beta diversity showed that samples from bulk tank milks were dissimilar but broadly clustered together (Fig. 2B). Farm bulk tanks were composed of a high number of genera that were present at low relative abundances of less than 5%. In one sample, these low-abundance genera accounted for 74.9% of the total population, and an average of 46.4% was seen across all bulk tank samples (Fig. 3; see also Fig. S1 and Table S1 in the supplemental material). There were 42 genera present at high relative abundances (>5% relative abundance in at least one sample) (Table S1), including traditionally milk-associated taxa such as *Pseudomonas* (mean, 6.6%), *Acinetobacter* (mean, 5.2%), *Lactococcus* (mean, 4.7%), *Corynebacterium* (mean, 4.2%), and *Streptococcus* (mean, 2.5%) (Fig. 3 and Table S1). In general, the microbial diversity of the bulk tank milks was such that the taxonomic compositions differed across farms and the two sampling days. However, it was apparent that the bulk tank milk profiles from some farms remained relatively more stable across the two sampling points; e.g., farm 23 had high relative abundances of *Leuconostoc* and *Acinetobacter* in both the mid- and late-lactation samples (Fig. 3). Overall, 17 high-relative-abundance genera, which were genera present at a >5% relative abundance in at least one sample, differed significantly in their relative abundances between paired mid- and late-lactation bulk tank samples (*P < *0.05). Nine genera showed higher relative abundances in mid-lactation bulk tanks, while eight were more abundant in late-lactation bulk tanks (Fig. 4). Among these, genera grouped as *Clostridium sensu stricto* subgroups 1 and 5 were both present at significantly higher relative abundances in the late-lactation bulk tank milks. Low-abundance genera, grouped as “others,” were also detected at significantly higher proportions in late-lactation bulk tanks (Fig. 4). One mid-lactation sample had a particularly high proportion of *Yersinia* (5.6%), leading to mid-lactation bulk tanks having, on average, a significantly higher relative abundance of this genus. There was also a larger number of genera (77) that were present at a lower relative abundance but at a >1% abundance in at least one sample, which significantly differed in abundance between mid- and late-lactation bulk tank samples (Fig. S2). It was again notable that taxa corresponding to the genus *Clostridium*, in this instance, *Clostridium sensu stricto* subgroups 15 and 18, were detected at significantly higher relative abundances in late-lactation samples.

10.1128/mSystems.00226-20.1 FIG S1 Relative abundances of genera present at a >1% relative abundance per sample. Shown are genus-level taxonomic classifications from 16S rRNA gene amplicon sequence data for mid- and late-lactation samples from bulk tanks (BT), collection tankers (CT), whole-milk silos (WMS), skimmed milk silos (SMS), skimmed milk powder (SMP), and cream (late lactation only). Genera shown are present at a >1% relative abundance in at least one sample. Download FIG S1, TIF file, 1.3 MB. Copyright © 2020 McHugh et al. 2020 McHugh et al. This content is distributed under the terms of the Creative Commons Attribution 4.0 International license.

10.1128/mSystems.00226-20.2 FIG S2 Significantly differential relative abundances of taxa constituting at least a 1% relative abundance in at least one sample between mid- and late-lactation bulk tanks. There were 77 significantly different taxa at a >1% relative abundance between mid- and late lactation. Download FIG S2, PDF file, 0.6 MB. Copyright © 2020 McHugh et al. 2020 McHugh et al. This content is distributed under the terms of the Creative Commons Attribution 4.0 International license.

10.1128/mSystems.00226-20.6 TABLE S1 Summary of the most abundant genera per group. Shown are 47 genera present at a >5% relative abundance in at least one sample and others (genera present at a <5% relative abundance), their mean relative abundances per group (± standard deviations [SD]), as well as their minimum and maximum relative abundances per group. Bold italic type indicates the group(s) in which the genera were present at a >5% relative abundance in at least one sample. Genera are listed in order of cumulative relative abundance for all samples (for each group, *n* = 67 for bulk tanks [BT], *n* = 11 for collection tankers [CT], *n* = 2 for whole-milk silos [WMS], *n* = 2 for skimmed milk silos [SMS], *n* = 9 for skimmed milk powder [SMP], and *n* = 1 for cream). Download Table S1, DOCX file, 0.05 MB. Copyright © 2020 McHugh et al. 2020 McHugh et al. This content is distributed under the terms of the Creative Commons Attribution 4.0 International license.

### Collection tanker milks retain relatively high microbial diversity but with some taxonomic convergence.

**FIG 5:**
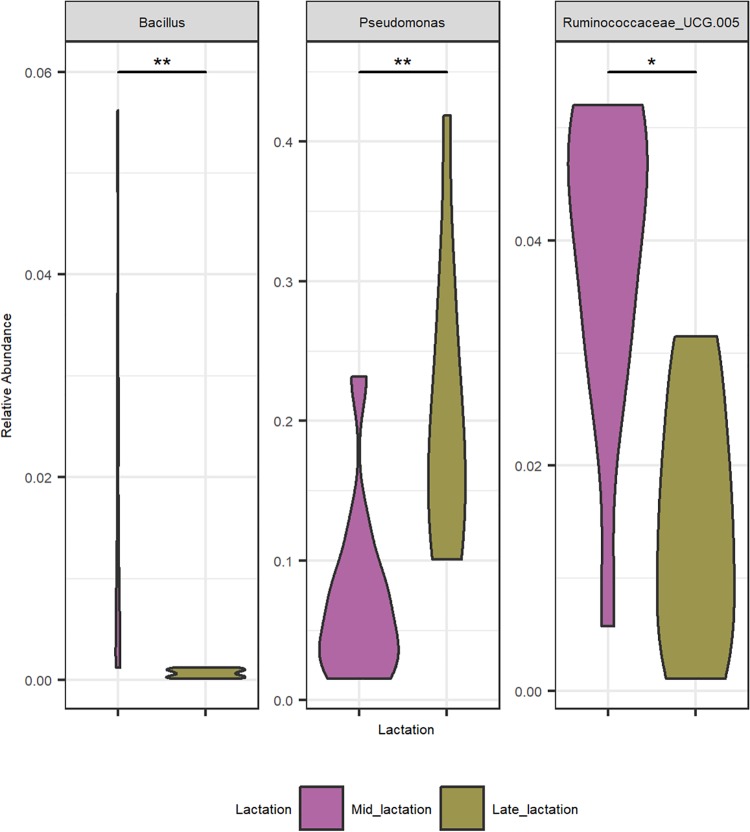
Significantly differential relative abundances of taxa constituting at least a 5% relative abundance in at least one sample between mid- and late-lactation collection tankers. Shown are violin plots of genera that differ significantly in relative abundances between mid- and late-lactation collection tankers. This highlights genera that are present at a >5% relative abundance in at least one sample. A pairwise Wilcoxon rank sum test using Benjamini-Hochberg *P* value correction/FDR correction analysis was performed, and samples are ordered by the lowest *P* value to the highest (**, *P < *0.01; *, *P < *0.05).

**FIG 6:**
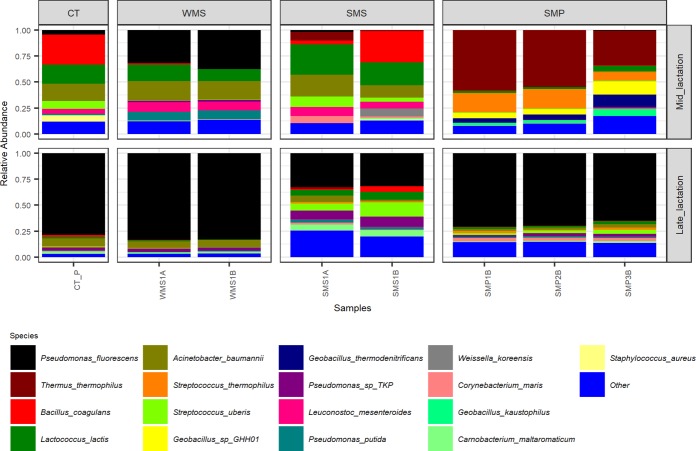
Relative abundances of species present at a >5% relative abundance in at least one sample. Shown are shotgun species-level taxonomic classifications from Kraken analysis using a filter threshold of 0.2 on a subset of samples from the mid- and late-lactation processing pipelines, including a pooled representative collection tanker (CT_P) sample, whole-milk silo (WMS) samples, skimmed milk silo (SMS) samples, and a subset of skimmed milk powder (SMP) samples from each lactation stage.

**FIG 7:**
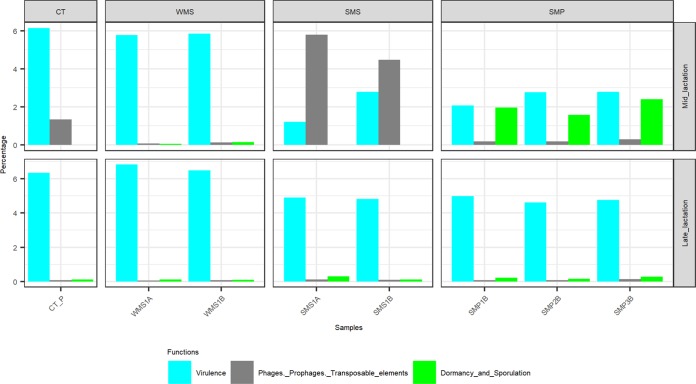
Percentages of a subset of functional classifications from SUPER-FOCUS L1 analysis attributed to prophage, phage, and transposable elements; dormancy and sporulation; as well as virulence in mid- and late-lactation pooled collection tanker (CT_P), whole-milk silo (WMS), skimmed milk silo (SMS), and skimmed milk powder (SMP) samples.

Milk samples obtained from collection tankers (CTs) were, with the exception of bulk tank milks, among those containing the highest microbial alpha diversity (Fig. 2A). Milk from mid-lactation collection tankers had a significantly higher microbial alpha diversity (*P < *0.05) than the corresponding late-lactation samples (Fig. 2A). From a beta diversity perspective, mid-lactation CT samples cluster closely with the bulk tanks from which they were filled (Fig. 2B). Late-lactation CT samples cluster further away from bulk tank samples, reflecting their collection on different days (as in the late-lactation period, the 67 farms from which bulk tank milk was collected did not yield a sufficiently large pool of milk to proceed with the powder manufacturing process) (Fig. 2B). Although the microbial composition of the tanker samples was diverse, and no two tankers had the same composition, a pattern of enrichment was apparent with respect to the taxa present at the highest relative abundances (Fig. 3 and Table S1), with *Acinetobacter*, *Pseudomonas*, *Lactococcus*, and *Corynebacterium* on average accounting for between 9% and 3% of the mid-lactation tanker milk microbiota composition (Fig. 3). These taxa were also present at high relative abundances in the previous bulk tank samples. Analysis of the corresponding samples collected during late lactation showed that although the tanker’s milk microbiota composition was diverse, *Pseudomonas* (mean, 20.3%) and *Acinetobacter* (mean, 25.4%) became the most dominant genera in each sample (Fig. 3 and Table S1). These were the genera with the highest relative abundances in bulk tank samples as well. Mid-lactation tanker milk samples had a higher proportion of low-abundance (<5%) and very-low-abundance (<1%) genera than the late-lactation tanker samples (Fig. 3 and Fig. S1). *Pseudomonas*, *Bacillus*, and *Ruminococcaceae* UCG.005 differed significantly between the mid- and late-lactation tanker samples, with *Pseudomonas*, or, more specifically, Pseudomonas fluorescens as determined by shotgun metagenomic sequencing, being present at a higher relative abundance in late-lactation samples. *Bacillus* and *Ruminococcaceae* UCG.005 were noted to be present at higher relative abundances in mid-lactation samples (Fig. 5). With respect to *Bacillus*, the pattern was driven by a higher relative abundance in mid-lactation tankers 4, 8, and 11 (Fig. 3 and Fig. S1). Shotgun metagenomic data established that at the species level, this taxon corresponded to Bacillus coagulans (Fig. 6). When SUPER-FOCUS was used to assign a functional classification to shotgun metagenomic reads (Fig. S3), higher relative abundances of virulence functions were noted in raw milks in pooled tanker samples than in heat-processed samples of skimmed milk silos (SMSs) or skimmed milk powders (Fig. 7). A high proportion of the shotgun metagenomic reads sequenced were not of microbial origin and were assigned to Bos taurus (Fig. S4), and so the resulting low number of microbe-associated reads did not allow strain-level classification or in-depth functional classification (Fig. S3). For this reason, the functional classifications with the most notable differences between groups are the only ones discussed (Fig. 7).

10.1128/mSystems.00226-20.3 FIG S3 SUPER-FOCUS L1 functional analysis. SUPER-FOCUS L1 functional analysis was performed on a subset of samples from the mid- and late-lactation processes, including a pooled representative collection tanker (CT_P) sample, whole-milk silo (WMS) samples, skimmed milk silo (SMS) samples, and a subset of skimmed milk powder (SMP) samples from each lactation stage. Download FIG S3, TIF file, 0.5 MB. Copyright © 2020 McHugh et al. 2020 McHugh et al. This content is distributed under the terms of the Creative Commons Attribution 4.0 International license.

10.1128/mSystems.00226-20.4 FIG S4 Percentage of Bos taurus aligned reads per shotgun metagenomic sample. There was a high percentage of Bos taurus aligned reads per shotgun metagenomic sample. Download FIG S4, TIF file, 0.3 MB. Copyright © 2020 McHugh et al. 2020 McHugh et al. This content is distributed under the terms of the Creative Commons Attribution 4.0 International license.

### Whole-milk silo storage results in an increased dominance of psychrotrophic spoilage-associated bacteria.

A decrease in microbial alpha diversity was observed in whole-milk silo (WMS) samples, regardless of the period of collection, compared to the corresponding samples from collection tankers (Fig. 2A). Beta diversity analysis displayed tight clustering of whole-milk silo samples, regardless of the lactation stage. Whole-milk silo samples cluster together, away from mid-lactation collection tankers and at the edge of the ellipse for late-lactation collection tankers (Fig. 2B). A high relative abundance of spoilage-associated psychrotrophic bacteria was observed in both mid- and late-lactation samples (Fig. 3), with *Pseudomonas*, *Acinetobacter*, as well as the nonpsychrotrophic *Lactococcus* genus dominating. These genera had begun to dominate the previous CT samples, particularly the late-lactation CT samples. Taxonomic compositions from mid- and late-lactation samples were similar (Fig. 3), *Leuconostoc* was the only genus present at a higher abundance in mid-lactation (mean, 6.3%) than in late-lactation (mean, 0.1%) samples (Table S1). In contrast, low-abundance genera, denoted “others,” were present at higher relative abundances in late-lactation (mean, 5.4%) than in mid-lactation (mean, 3.1%) samples (Table S1). However, all WMS samples had a smaller proportion of low-abundance genera than the collection tankers and bulk tank milks (Fig. 3). This reflected the lower alpha diversity of these samples than those collected earlier in the milk processing chain (Fig. 2A). WMS samples contained no unique genera at a >5% relative abundance that had not been noted in previous BT and CT samples (Table S1 and Fig. S5). Shotgun metagenomic analysis revealed species-level classifications of the most abundant genera across all WMS samples as Pseudomonas fluorescens, Acinetobacter baumannii, and Lactococcus lactis (Fig. 6); this was in agreement with 16S genus-level classifications. Due to the high proportion of shotgun metagenomic reads assigned to Bos taurus (Fig. S4), the resulting low number of bacterium-associated reads did not allow strain-level classification. When SUPER-FOCUS was used to assign a functional classification to the shotgun metagenomic reads (Fig. S3), a higher relative abundance of virulence functions was noted for raw milks in the WMS and pooled tankers than for all other samples (Fig. 7).

10.1128/mSystems.00226-20.5 FIG S5 Venn diagram showing genera at a >5% relative abundance at each stage of processing. Shown is the overlap of taxa across different sample types during mid- and late lactation. Included are bulk tanks (BT), collection tankers (CT), whole-milk silos (WMS), skimmed-milk silos (SMS), skimmed milk powder (SMP), and cream. BT* reflects the collection of late-lactation BT samples at a different time from the other late-lactation samples. Seven BT* taxa are shared with late CT and SMP samples. *P*, *Pseudomonas*; *Ac*, *Acinetobacter*; *Lc*, *Lactococcus*; *Str*, *Streptococcus*; *Sta*, *Staphylococcus*; *Ge*, *Geobacillus*; *Psy*, *Psychrobacter*; *Ar*, *Arthrobacter*; *Ba*, *Bacteroides*; *Le*, *Leuconostoc*; *Th*, *Thermus*; *Ko*, *Kocuria*; *AeC*, *Aerococcus*; *Baci*, *Bacillus*; *Je*, *Jeotgalicoccus*; *Lb*, *Lactobacillus*; *Pa*, *Paracoccus*; *Rh*, *Rhodococcus*; *Mo*, *Moraxella*; *Ma*, *Macrococcus*; *Ro*, *Rothia*; *Bi*, *Bifidobacterium*; *Fl*, *Flavobacterium*; *Br*, *Brachybacterium*; *En*, *Enterococcus*; *Pc*, *Pseudoclavibacter*; *Cl*, *Clavibacter*; *Ca*, *Carnobacterium*; *At*, *Atopococcus*; *AeM*, *Aeromonas*; *An*, *Anoxybacillus*; *Em*, *Empedobacter*; *Ex*, *Exiguobacterium*; *Po*, *Porphyromonas*; *Ye*, *Yersinia*; *Bro*, *Brochothrix*; *Ha*, *Hafnia*; *Co*, *Corynebacterium* 1; *Ru*, *Ruminococcaceae* UCG.005; *Chri*, *Christensenellaceae* R.7 group; *Chry*, *Chryseobacterium*; *Pe*, *Peptoclostridium*; *Cl1*, *Clostridium sensu stricto* 1; *Cl5*, *Clostridium sensu stricto* 5; *uF*, uncultured *Fusobacterium* sp.; *ub*, uncultured bacterium; *St*, *Stenotrophomonas.* Download FIG S5, TIF file, 0.5 MB. Copyright © 2020 McHugh et al. 2020 McHugh et al. This content is distributed under the terms of the Creative Commons Attribution 4.0 International license.

### The skimmed milk silo microbial composition differs across samples collected in different seasons.

The alpha diversity of the skimmed milk silo (SMS) microbiota was higher than that of the preceding whole-milk silo samples (Fig. 2A). Beta diversity analysis showed that the microbiota of mid-lactation SMS samples clustered separately from WMS samples. In contrast, late-lactation samples clustered closely with their corresponding WMS samples (Fig. 2B). From a taxonomic perspective, the relative abundance of the dominant psychrotrophic bacteria detected at the preceding WMS stage was reduced in the mid-lactation SMS samples following pasteurization and separation of that milk (Fig. 3). This decrease was not as evident in late-lactation samples, with *Pseudomonas*, *Lactococcus*, and *Acinetobacter* remaining dominant (Fig. 3). SMS samples from both time points contained *Lactococcus*, *Acinetobacter*, and *Streptococcus* (Fig. 3); the former two genera were present at high relative abundances at all previous processing stages, whereas the latter, *Streptococcus*, was present at a high relative abundance in the preceding BT and CT samples but not in the WMS samples. Species-level taxonomic analysis revealed that these primarily corresponded to Lactococcus lactis, Acinetobacter baumannii, Streptococcus thermophilus, and Streptococcus uberis at the species level (Fig. 6). Mid-lactation SMS samples had a higher proportion of low- and very-low-abundance bacteria than the late-lactation SMS samples (Fig. 3, Table S1, and Fig. S1). When SUPER-FOCUS was used for the functional classification of shotgun metagenomic reads (Fig. S3), higher relative abundances of genes associated with phage, prophage, and transposable elements were noted in mid-lactation SMS samples than in all other samples (Fig. 7).

### The cream silo microbiota differs from those of other dairy processing samples.

The late-lactation processing pipeline provided the only cream sample available for analysis within this study. This sample had a higher alpha diversity than the whole-milk silo sample from which it was produced and the skimmed milk silo contents from which it was separated (Fig. 2A). The microbial communities in the sample did not cluster with either the late-lactation skimmed milk silo or the whole-milk silo samples (Fig. 2B). This sample was dominated by *Anoxybacillus* (Fig. 3), a genus present at a <1% relative abundance at previous stages of the processing pipeline, as well as *Lactococcus*, *Corynebacterium*, and *Acinetobacter*, among other genera (Fig. 3 and Table S1). Low-abundance genera accounted for 18.0% of the cream silo microbiota (Table S1), and 6.4% of the microbiota corresponded to very-low-abundance genera.

### The dairy powder microbiota can vary, reflecting either the original raw-milk microbiota or microbes selected for during processing.

The microbiotas of skimmed milk powder (SMP) samples differed in a manner that reflected the impact of processing on particular days. The microbiota of mid-lactation skimmed milk powder samples had a lower alpha diversity than that of the SMS milk from which it was produced (Fig. 2A). Furthermore, the microbial communities in the mid-lactation skimmed milk powders clustered separately from both the late-lactation skimmed milk powder samples and the milks from which they were derived (Fig. 2B). More specifically, the mid-lactation powders showed a shift in microbial taxonomic dominance, with thermophilic bacteria such as *Thermus*, *Geobacillus*, and *Streptococcus* being more dominant in these samples (Fig. 3). Notably, *Thermus* and *Geobacillus* had not been seen in any previous stages of the processing pipeline. Shotgun metagenomic sequencing assigned these as Thermus thermophilus, *Geobacillus* sp. strain GHH01, Geobacillus thermodenitrificans, and Streptococcus thermophilus. SUPER-FOCUS functional classification highlighted corresponding increases in the relative abundances of sporulation- and dormancy-associated genes in mid-lactation skimmed milk powder samples (Fig. 7), reflecting the proportions of spore-forming bacteria present. In these samples, low-relative-abundance genera (<5%) accounted for 0.9% to 2.9% of reads, and very-low-abundance genera (<1%) accounted for 0.45% to 1.3% of reads. The microbiotas of late-lactation skimmed milk powders differed considerably. An increase in diversity was observed in late-lactation skimmed milk powders compared to the SMS milk from which they were produced (Fig. 2A), and these samples clustered closely to the skimmed milk samples from which they were derived (Fig. 2B). Taxonomic analysis revealed the dominance of psychrotrophic genera and, more specifically, of the same genera and species (P. fluorescens and A. baumannii) that had dominated previous WMS and SMS samples (Fig. 3 and 5). Late-lactation powder had significantly higher (*P < *0.01) microbial alpha diversity than the mid-lactation powder (Fig. 2A).

### Sample source and lactation stage significantly influence differences between the microbiota of the dairy samples.

Overall, Adonis analysis from the R vegan package for Bray-Curtis beta diversity analysis showed significant differences between samples based on the lactation stage and source of the sample (*P* ≤ 0.001), Twenty percent of the variation in the distance between samples was attributed to the sample source (BT/CT/WMS/SMS/SMP/cream), and 9.5% of the variation in the distance between samples was attributed to the lactation stage (ML, late lactation 1 [LL1], and LL2), with 4.7% being due to both the source and lactation stage.

## DISCUSSION

**FIG 8:**
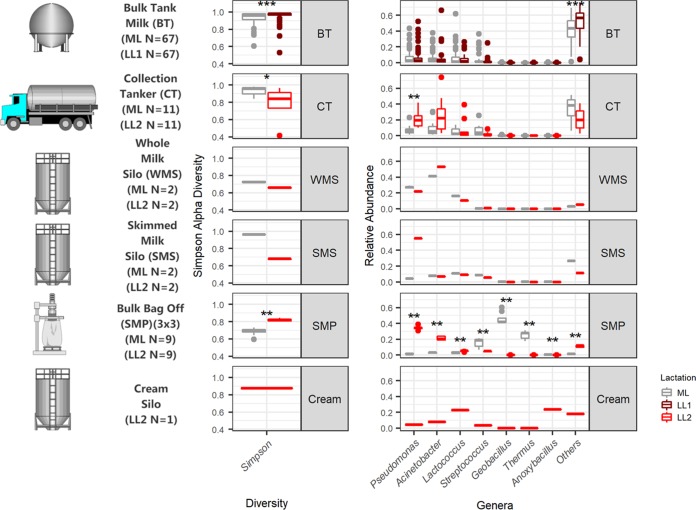
Summary of differences in results due to sampling location and lactation stage. A brief sampling schematic with numbers, Simpson alpha diversity values, as well as genera with an average of at least a 10% relative abundance per sample location and lactation stage are shown, and significant differences are highlighted (***, *P < *0.001; **, *P < *0.01; *, *P < *0.05).

This study set out to use molecular methods to provide an important description of the microbiota of a food processing pipeline by tracking the microbiota of raw milks on farms to a final skimmed milk powder. Through HTS, it was demonstrated that different production days and microbial selection by processing parameters can impact the microbiota during this process (Fig. 8). Through this approach, this study expands upon previous investigations (14, 19–21) to give an even greater understanding of the changes in the dairy microbiota from milk to skimmed milk powder. This investigation confirms the diversity of the raw-milk microbiota; however, it highlights that bulk tank samples from mid- and late lactation broadly cluster together. Upon bulk refrigerated storage, there is a shift toward a psychrotolerance-dominant, processing facility-selected community that is dominated by *Pseudomonas*, *Acinetobacter*, and *Lactococcus*. This is consistent with a U.S. study by Kable et al. (14), which noted that large-scale silo storage led to the convergence of the microbiota into one of two community types. The subsequent fate of these bacteria following further product processing in our study showed that the relative abundances of these dominant psychrotrophic bacteria diminished following pasteurization. This was particularly apparent in mid-lactation samples, possibly due to a number of reasons not limited to the lactation stage and associated warmer weather, with an increase in thermophilic bacteria during the processing of these samples, with colder weather and lower production rates associated with late-lactation, psychrotroph-dominant samples. Continued processing of mid-lactation samples to a skimmed milk powder had a considerable impact on the microbiota. The resulting powder was dominated by the thermophiles T. thermophilus and *Geobacillus* sp. This dominance may be due to a number of factors, including, but not limited to, contamination from within the processing facility (5, 22) and/or their enrichment due to high-temperature treatment in SMP processing as well at the fact that the samples were collected from processing during a warmer time of year and when powder production was at its peak, with equipment running for longer periods and with higher throughput. The presence of T. thermophilus is notable as it was previously detected in environmental samples from a cheese manufacturing facility and has been shown to have a pinking defect in continental-type cheeses (18). *Geobacillus* is a high-heat-resistant thermophilic sporeformer known to contaminate dairy powders. The high heat in SMP manufacture allows *Geobacillus* to thrive when other microorganisms succumb to high-heat treatment, and its spore-forming ability facilitates subsequent survival in powdered dairy.

In contrast to mid-lactation samples, the microbiota of late-lactation samples did not change as considerably throughout processing. It is not clear if this was due to seasonality or potential process-related factors such as differences in cleaning practices used at the later sampling time point. It may also be that thermophilic or desiccation-tolerant bacteria were absent, or were present at only low levels, in the raw ingredients or processing system to take advantage of the high heat and drying in SMP processing on this processing day. Overall, significant differences between samples were accounted for by both the source of the sample and the lactation period. Ultimately, as this was the first such comprehensive study of its kind, further investigation is needed to determine how or why thermophilic species were selected for in one processing run on 1 day in a mid-lactation period and were not selected for in a processing run on 1 day in a late-lactation period. An increased understanding of why and how these differences occurred will undoubtedly aid dairy processors globally to implement effective measures and decrease undesirable microorganisms while increasing food safety and quality.

Although no previously undetected or unexpected taxa were identified, a number of potentially pathogenic bacteria were detected in the samples. One mid-lactation bulk tank sample in isolation had a high (>5%) relative abundance of *Yersinia*; however, this accounted for <0.4% of all processed samples, suggesting its elimination by heat treatments and processing. A number of *Clostridium sensu stricto* subgroups were determined to be present at significantly higher relative abundances in late lactation than in mid-lactation bulk tanks; however, the relative abundances of these subgroups also decreased following transport and processing. SUPER-FOCUS functional gene classification noted higher relative abundances of sporulation- and dormancy-associated functional gene groups in mid-lactation skimmed milk powder samples containing thermophilic spore-forming *Geobacillus* species than in all other samples. Although *Geobacillus* species are not pathogenic, they are extremely difficult to eliminate from the processing environment due to their heat tolerance and their ability to form resistant spores and biofilms, which can harbor pathogenic species (23, 24). A. baumannii was determined by shotgun metagenomic sequencing to be the dominant species of *Acinetobacter*. Although traditionally associated with opportunistic infections, drug resistance, and nosocomial infections, A. baumannii has been detected in animal products and was shown to have epidemiological characteristics that are different from those of strains that cause nosocomial infections (25). It has also been detected in bulk tank milks and dairy powders, leading the Food and Agriculture Organization of the United Nations (FAO) to classify it as one of the “category B organisms—causality plausible, but not yet demonstrated,” with respect to causing infant illness from powdered infant formula (26). The low number of shotgun metagenomic reads did not allow accurate strain-level identification or the identification of specific virulence-associated genes to facilitate a more in-depth investigation. Despite this, SUPER-FOCUS functional gene classification showed higher relative abundances of virulence-associated functional gene groups in raw-milk samples in tankers and whole-milk silos than in heat-processed samples in skimmed milk silos and skimmed milk powders. Overall, there is a general pattern of a greater relative abundance of potentially pathogenic genera and species in raw-milk samples and an increased relative abundance of spore-forming species present in mid-lactation skimmed milk powders. However, it should be emphasized that relative abundances are reported throughout this paper, and further analysis would be needed to confirm if there were increases and decreases in absolute abundances. It should be noted that the relative-abundance results obtained largely agree with data from corresponding culture analyses (27).

This study highlights that changes in the dairy microbiota throughout processing vary across processing days. It highlights the merits of using HTS to monitor processing-facility-induced changes in the dairy microbiota and potential future benefits of applying this technology more specifically to elucidate the basis for differences in dairy powder composition. Species-level composition analysis from shotgun metagenomic sequencing enables a more in-depth analysis than previously possible with 16S rRNA gene amplicon sequencing. Further development of this method opens the possibilities of routine microbiology testing, improved detection of sources of contamination, tracking of microorganisms throughout the food chain, and, in general, enhancing the ability of processors to make informed decisions to reduce risk and waste. There are a number of ways in which the approach taken in this study can be improved upon for commercial applications, such as combining it with quantitative approaches to obtain absolute numbers. Untargeted shotgun analysis can be subject to host DNA contamination (28), and dairy samples can have particularly high levels of host DNA, as somatic cell counts can be high in raw dairy. This can result in decreased yields of microbial DNA sequence, thereby limiting the number of reads available for comprehensive microbial analysis (see Fig. S4 in the supplemental material). The removal of host DNA prior to sequencing is possible by utilizing microbiome enrichment kits (29) and can be considered for performing a similar analysis in the future. However, this approach increases the overall cost. Another approach could be to enrich for specific subpopulations prior to DNA extraction (30). Developments such as these, combined with advances in portable sequencing technologies and further advances in the speed and accuracy of *in silico* tools, have the potential to greatly assist decision-making in this and other food chains.

### Conclusions.

In conclusion, this study provides detailed insight into the changes in the microbiota of dairy samples throughout a milk powder manufacturing process, on distinct sampling days. A notable change was observed upon large-volume pooling in the processing facility, which resulted in the dairy microbiota becoming dominated by psychrotolerant, spoilage-associated bacteria. Also of note were the impacts of processing and the processing facility on the microbiota. A pattern of particular note is that low levels of thermophilic bacteria present in raw ingredients, or within the processing facility, can potentially proliferate in the absence of competitors during and following processing and dominate the processed dairy product. With the routine implementation of these methods, an understanding of the reasons that lead to different species being dominant in the final product can be determined and lead to informed decisions regarding product fate, in turn leading to increased food safety, reduced risk, and reduced economic losses.

## MATERIALS AND METHODS

### Sample collection.

Raw milks, pasteurized milks, and powdered dairy products were sampled from within a commercial milk processing pipeline (Fig. 1). Raw-milk bulk tanks were sampled on one day during the early- to mid-lactation period (May 2016), here referred to as mid-lactation. These milks were combined and further processed to a skimmed milk powder. During this process, samples were also collected from collection tankers, the processor’s whole-milk silo, the skimmed milk silo, and the resultant skimmed milk powders. Samples from raw-milk bulk tanks (October 2016) and the processing pipeline (December 2016), which in this instance also included sampling of the cream silo, were also collected later in the year to represent the late-lactation period. In order to complete the full process, a minimum capacity of milk was required, and considering the lower rate of herd production in late lactation, a greater number of farm bulk tanks was required. Therefore, during the late-lactation period, the original farms were resampled separately from the process (collection tanker to skimmed milk powder), which contained different farm bulk tank inputs (*n* = 150, not sampled). Samples were collected by personnel at the processing facility using standard collection procedures, and liquid samples were transported on ice to the laboratory, where DNA extraction was performed immediately. Skimmed milk powders were transported at room temperature and stored for up to 1 month prior to DNA extraction.

### DNA extraction.

DNA was extracted from fresh liquid dairy samples. Powders were stored at room temperature, and upon reconstitution at 10% (wt/vol) in one-quarter-strength Ringer’s solution, DNA was extracted from 30-ml milk samples and 50-ml reconstituted skimmed milk powder samples using the MoBio PowerFood DNA isolation kit according to the manufacturer’s instructions, with some minor adjustments. Samples were centrifuged at 5,000 × *g* for 20 min at 4°C. Fat was removed (from raw-milk samples), and the supernatant was discarded. From there, pellets were washed and subjected to lysozyme treatment as previously reported (30). Twenty-eight microliters of proteinase K was added, and the mixture was incubated at 55°C for 15 min. Samples were centrifuged at 13,000 × *g* for 1 min, and the supernatant was discarded. Pellets were resuspended in 450 μl PF1 solution from the PowerFood kit, and from this point, the kit protocol was followed, including the recommended alternative lysis step for difficult-to-lyse cells. DNA was eluted in 50 μl of elution buffer.

### 16S rRNA amplicon sequencing.

Template DNA was quantified and checked for quality by using the Qubit double-stranded DNA (dsDNA) high-sensitivity assay kit as well as by running 2 μl on a 1% agarose gel. DNA was normalized to 5 ng μl^−1^. The V3-V4 variable region of the 16S rRNA genes was amplified in triplicate from each sample as previously described (7), with a few changes: 35 PCR cycles were used instead of 32, and Kapa2G Robust (Kapa Biosystems Ltd.) was used instead of Kapa HiFi Hotstart. Two microliters of each PCR mixture was run on a 1% agarose gel to check for quality before pooling triplicate PCR mixtures, with cleaning with a 0.8× volume of AMPure XP beads. Fifty microliters of the cleaned-up sample was stored at −20°C. Five microliters was aliquoted and subjected to index PCR and cleanup according to Illumina 16S metagenomic sequencing library preparation guidelines, as previously described (7). DNA concentrations were quantified using the Qubit dsDNA high-sensitivity assay kit before diluting to 20 nM, pooling, and performing a final 1:1 AMPure XP cleanup step. The samples were pooled into 4 pools. Samples from each processing step were contained in each pool, with mid-lactation skimmed milk powder samples included in each pool as a control. The samples were sequenced on the Illumina MiSeq sequencing platform in the Teagasc sequencing facility using a 2-by-250 V2 kit according to Illumina sequencing protocols.

### Whole-metagenome shotgun sequencing.

A subset of samples was selected for whole-metagenome shotgun sequencing. These included 4 WMS (2 mid-lactation and 2 late lactation), 4 SMS (2 mid-lactation and 2 late lactation), 6 SMP (3 mid-lactation and 3 late lactation [1 from each bag]), as well as 2 pooled collection tanker (CT_P) samples, in which equal volumes of DNA from each of the 11 mid-lactation samples were pooled into one sample and equal volumes of each of the late-lactation tankers were pooled into a second sample. Samples were prepared according to the Nextera XT DNA library preparation guide from Illumina. Samples were sequenced on the Illumina MiSeq sequencing platform at the Teagasc sequencing facility with a 2-by-250 V2 kit with standard Illumina sequencing protocols.

### Bioinformatic and statistical analyses.

16S rRNA gene amplicon sequences were processed as previously described (7). Briefly, forward and reverse reads were joined using USEARCH FLASH (fast length adjustment of short reads to improve genome assemblies) (31). Paired-end reads were further processed by quality filtering based on a quality score of 25 and removing mismatched barcodes and sequences below length thresholds by QIIME (32). USEARCH v7 (64-bit) (33) was utilized for removing noisy data, detecting chimeras, and clustering into operational taxonomic units (OTUs) at 97% identity. OTUs were aligned using PyNAST (Python Nearest Alignment Space Termination) (a flexible tool for aligning sequences to a template alignment [34]), and taxonomy was assigned using BLAST (35) against the SILVA SSURef database, release 123 (36). QIIME data were further analyzed using Phyloseq in R (37), richness was estimated to obtain alpha diversity, and distances were obtained for beta diversity before visualization using ggplot2 (38). Taxonomy was also visualized using ggplot2. A pairwise Wilcoxon rank sum test using Benjamini-Hochberg *P* value correction/false discovery rate (FDR) correction analysis was used to compared sample groups from mid- and late lactation. Adonis from the R vegan package was used to determine differences in beta diversity. Shotgun metagenomic data were processed as previously described (30). Briefly, raw metagenomic reads were checked for the presence of bovine reads, which were removed; filtered based on the presence of quality and quantity; and trimmed to 170 bp with a combination of Picard tools and SAMtools (39). Kraken with a filter threshold of 0.2 (40) and SUPER-FOCUS (41) were used to determine microbial composition to the species level and biological functions, respectively.

### Data availability.

All sequence data have been deposited in the European Nucleotide Archive (ENA) under study accession number PRJEB31110.
