# Supplementary figures and images for: Tracking the Dairy Microbiota from Farm Bulk Tank to Skimmed Milk Powder
Source: mSystems. 2020 Apr 7;5(2):e00226-20. doi: 10.1128/mSystems.00226-20 (PMC7141888; doi:10.1128/mSystems.00226-20)

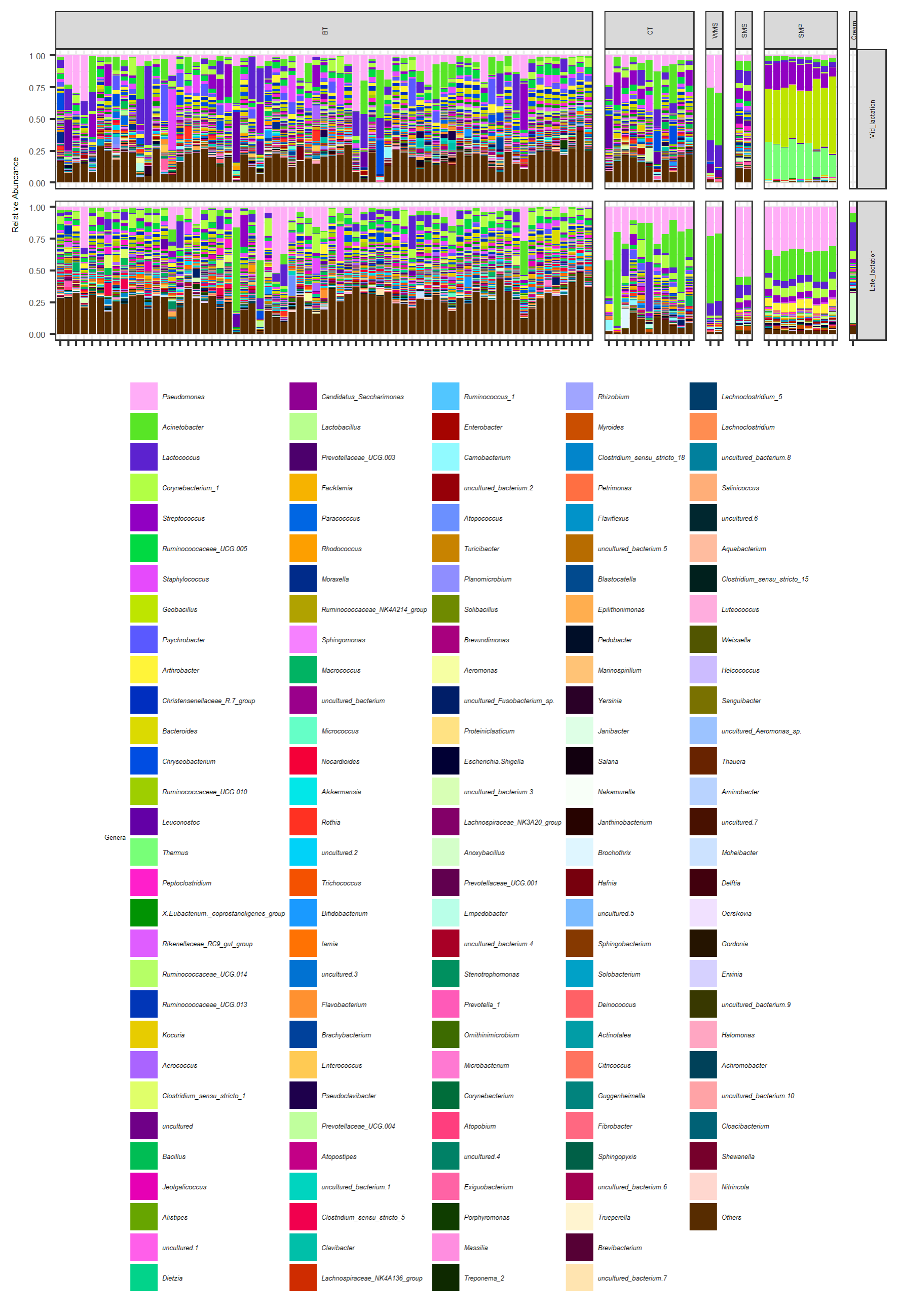

Supplement: FIG S1 [file mSystems.00226-20-sf001.tif]

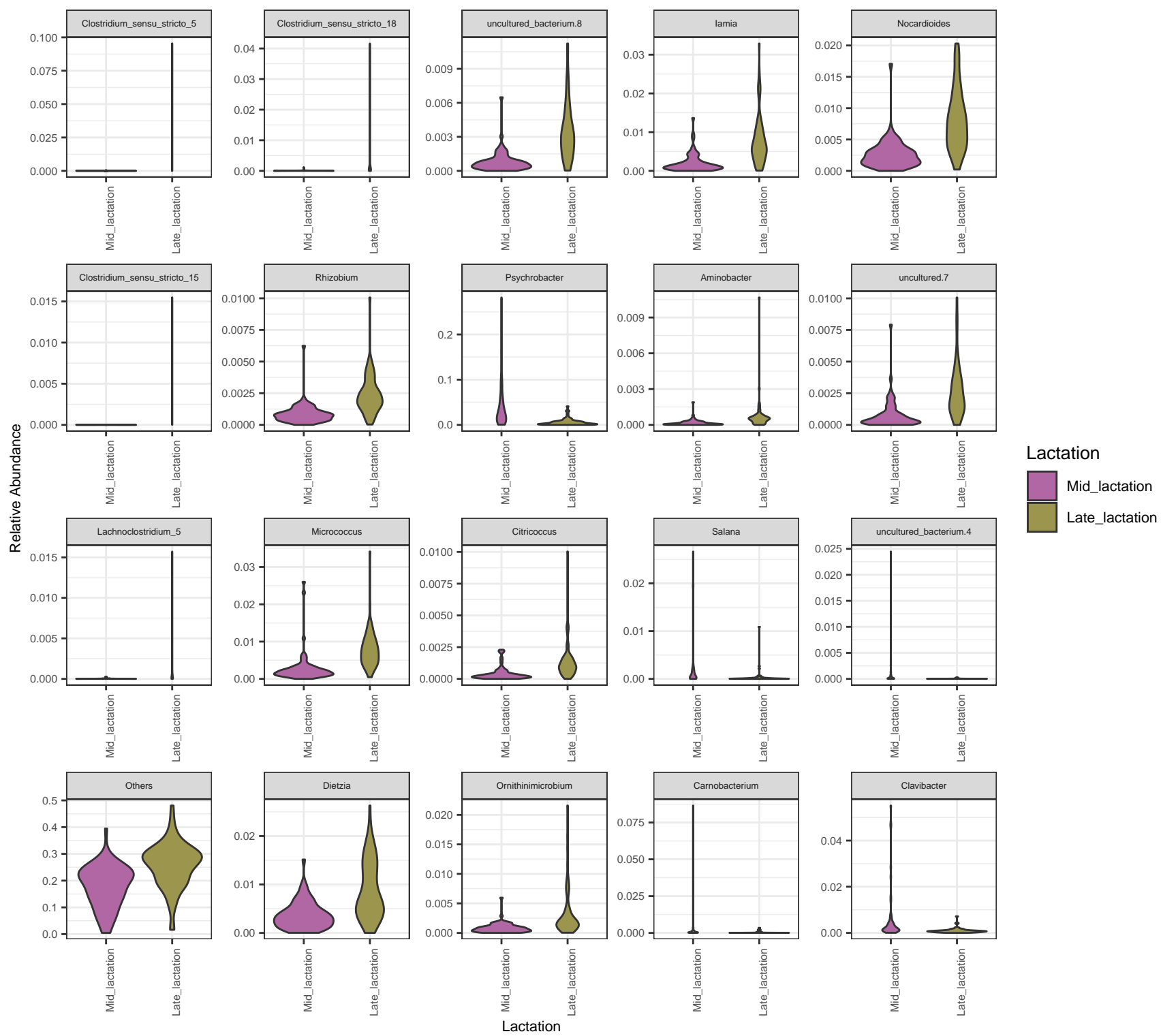

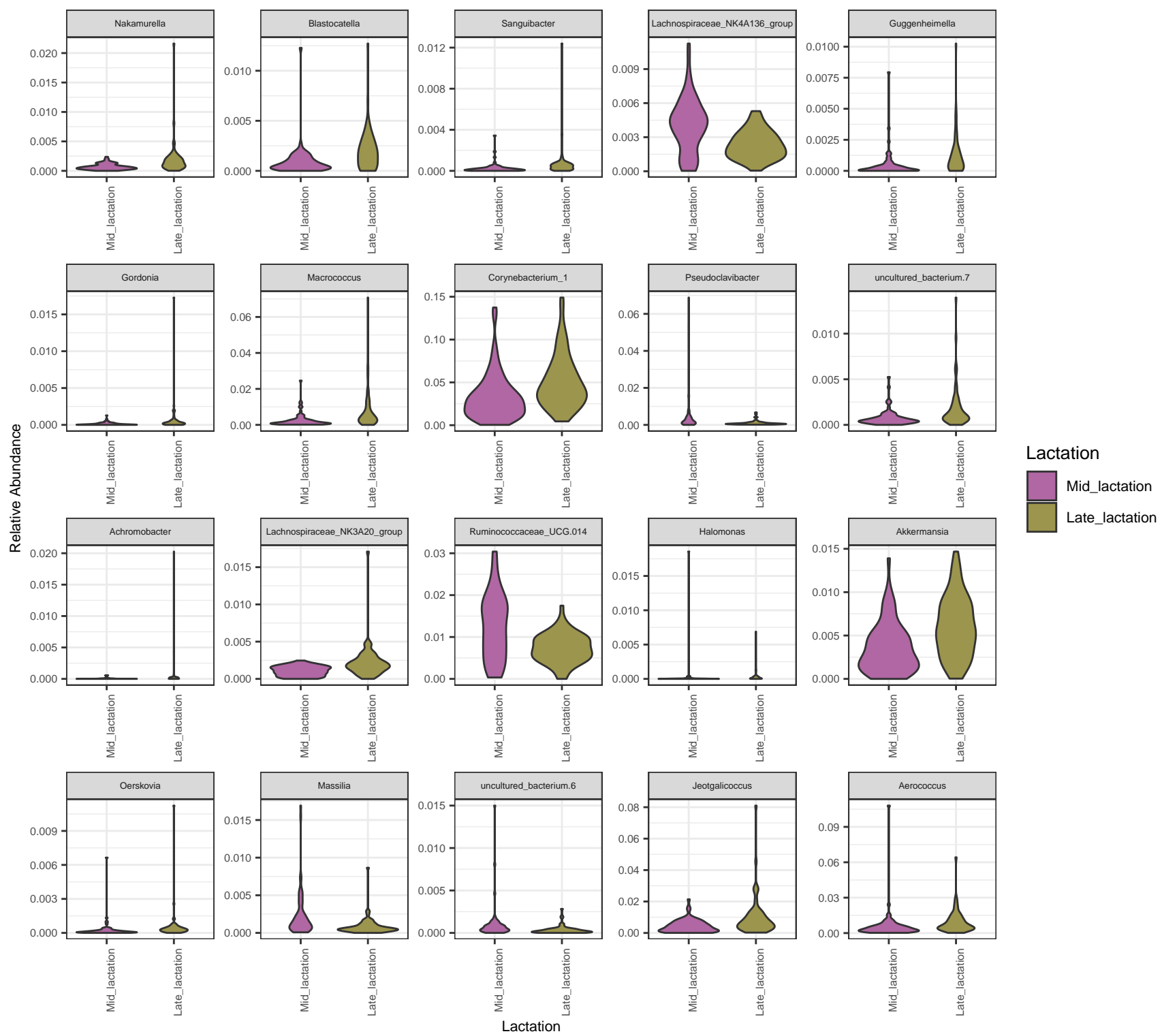

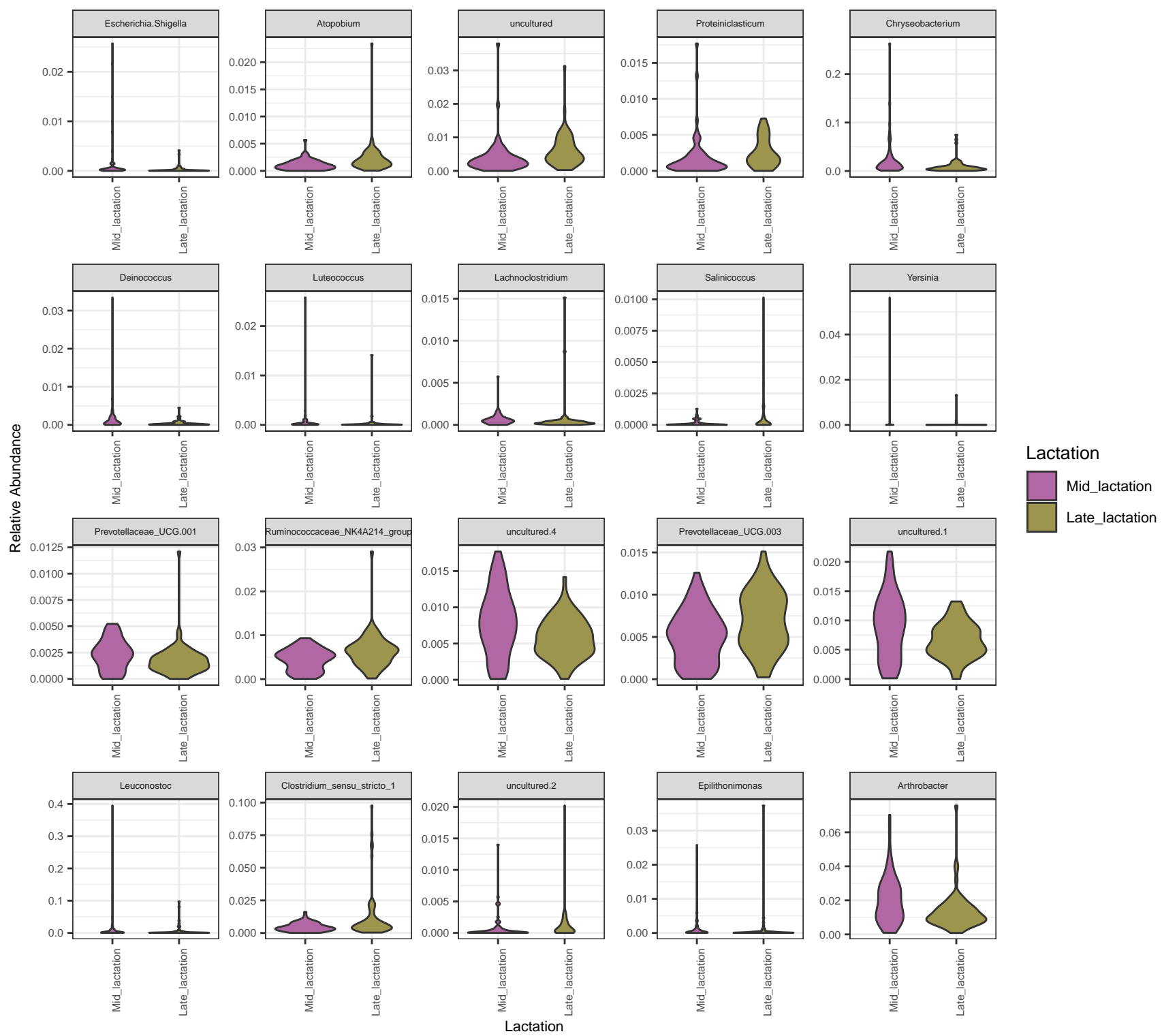

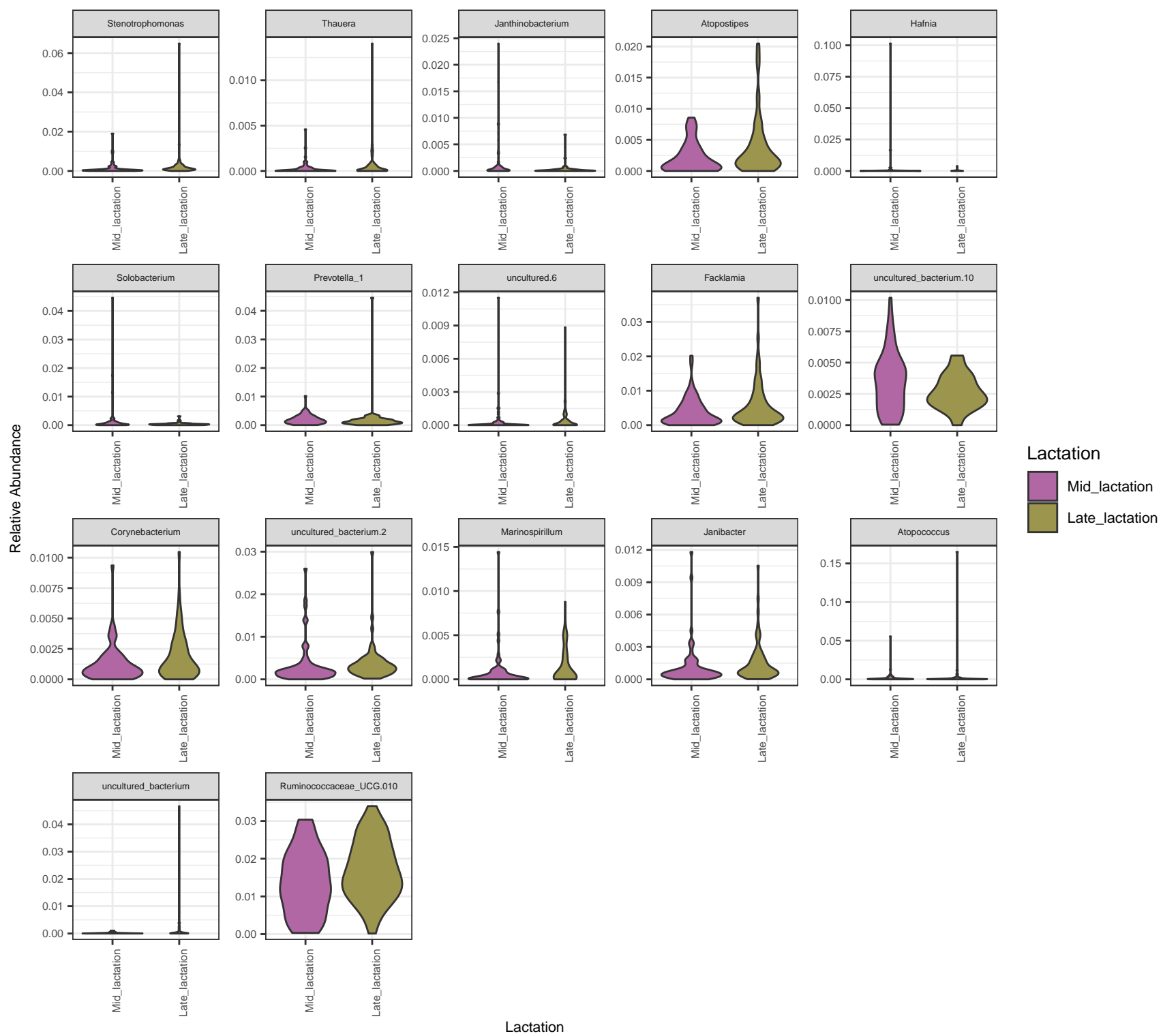

Supplement: FIG S2 [file mSystems.00226-20-sf002.pdf]

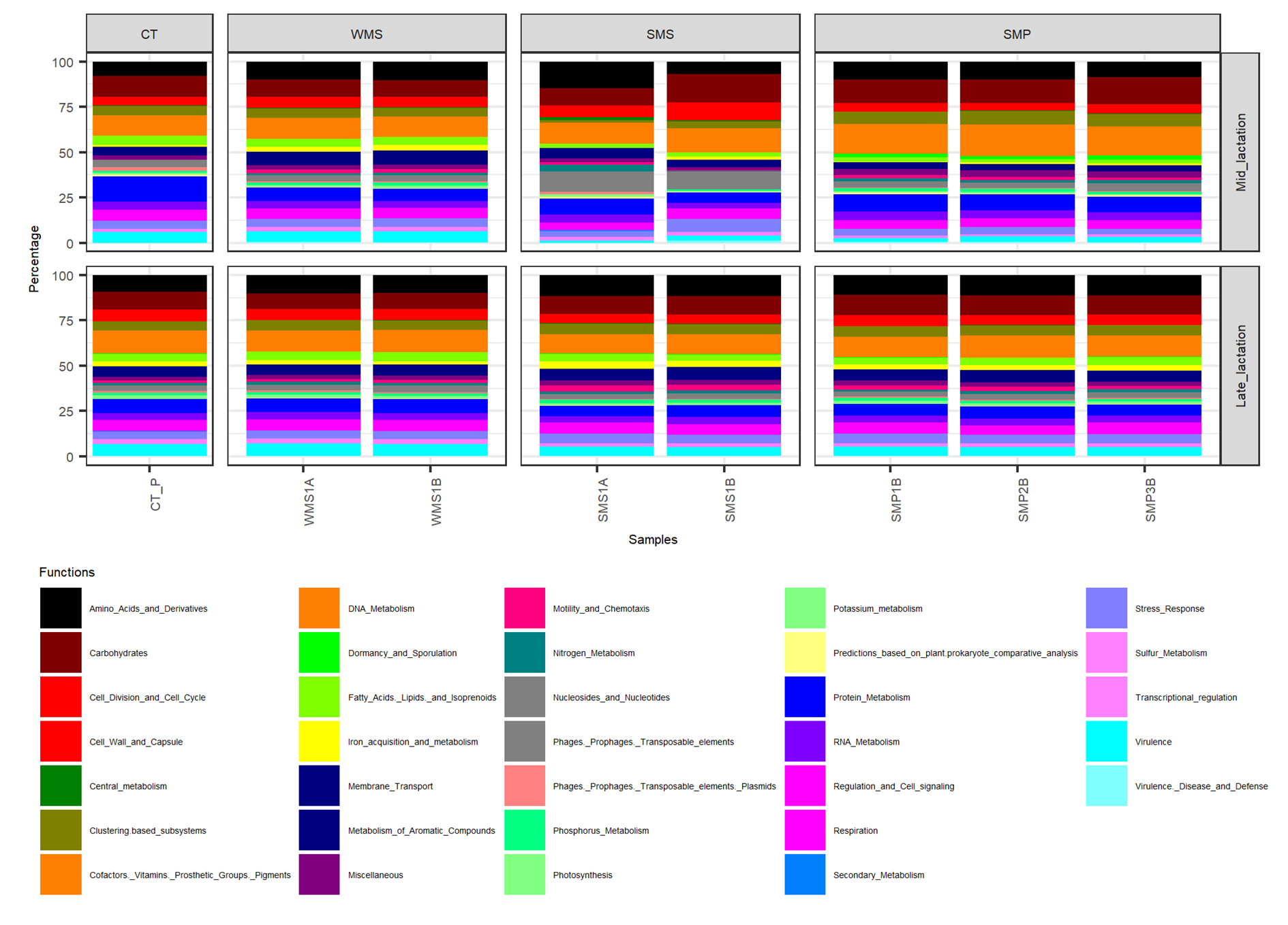

Supplement: FIG S3 [file mSystems.00226-20-sf003.tif]

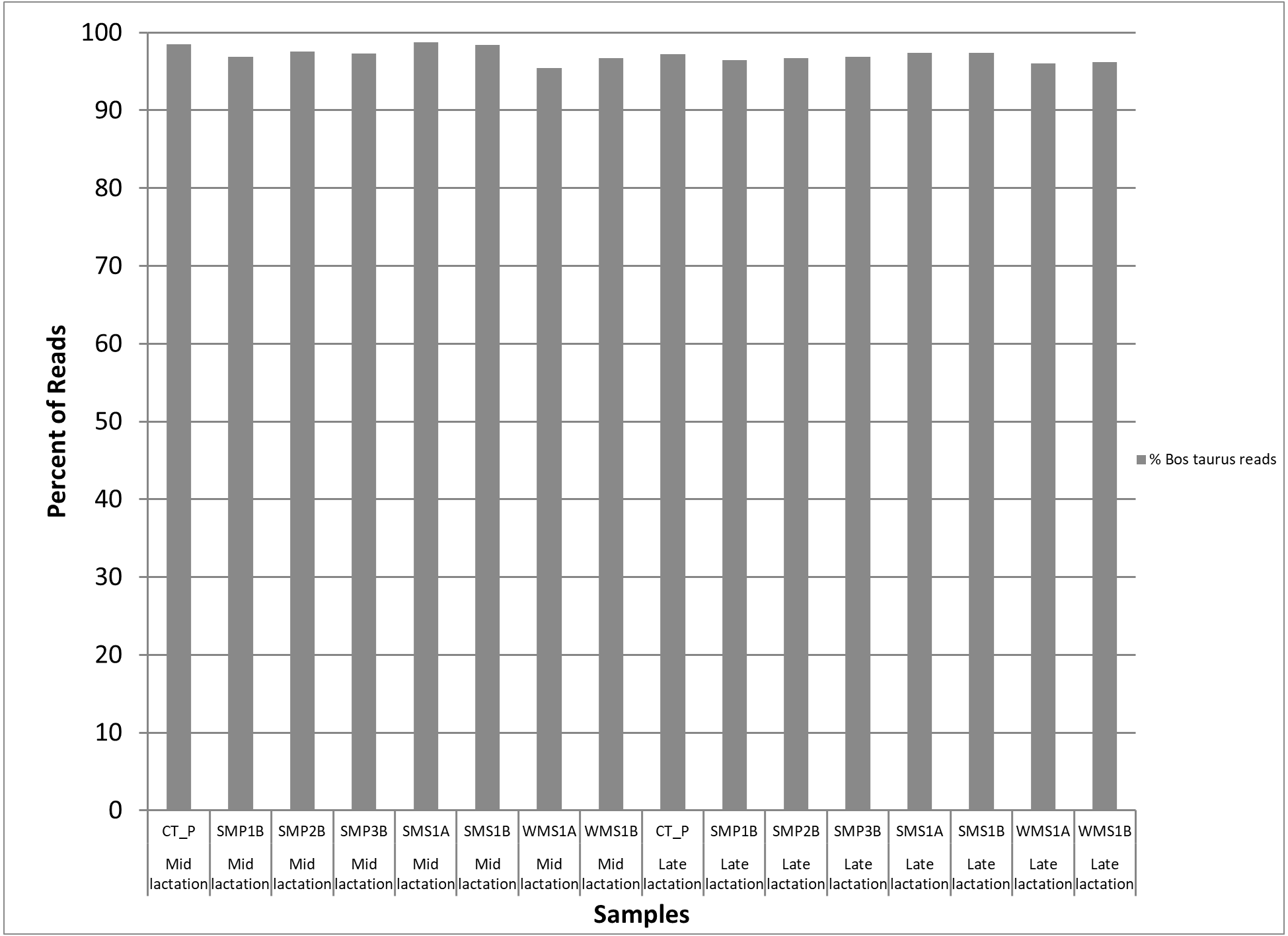

Supplement: FIG S4 [file mSystems.00226-20-sf004.tif]

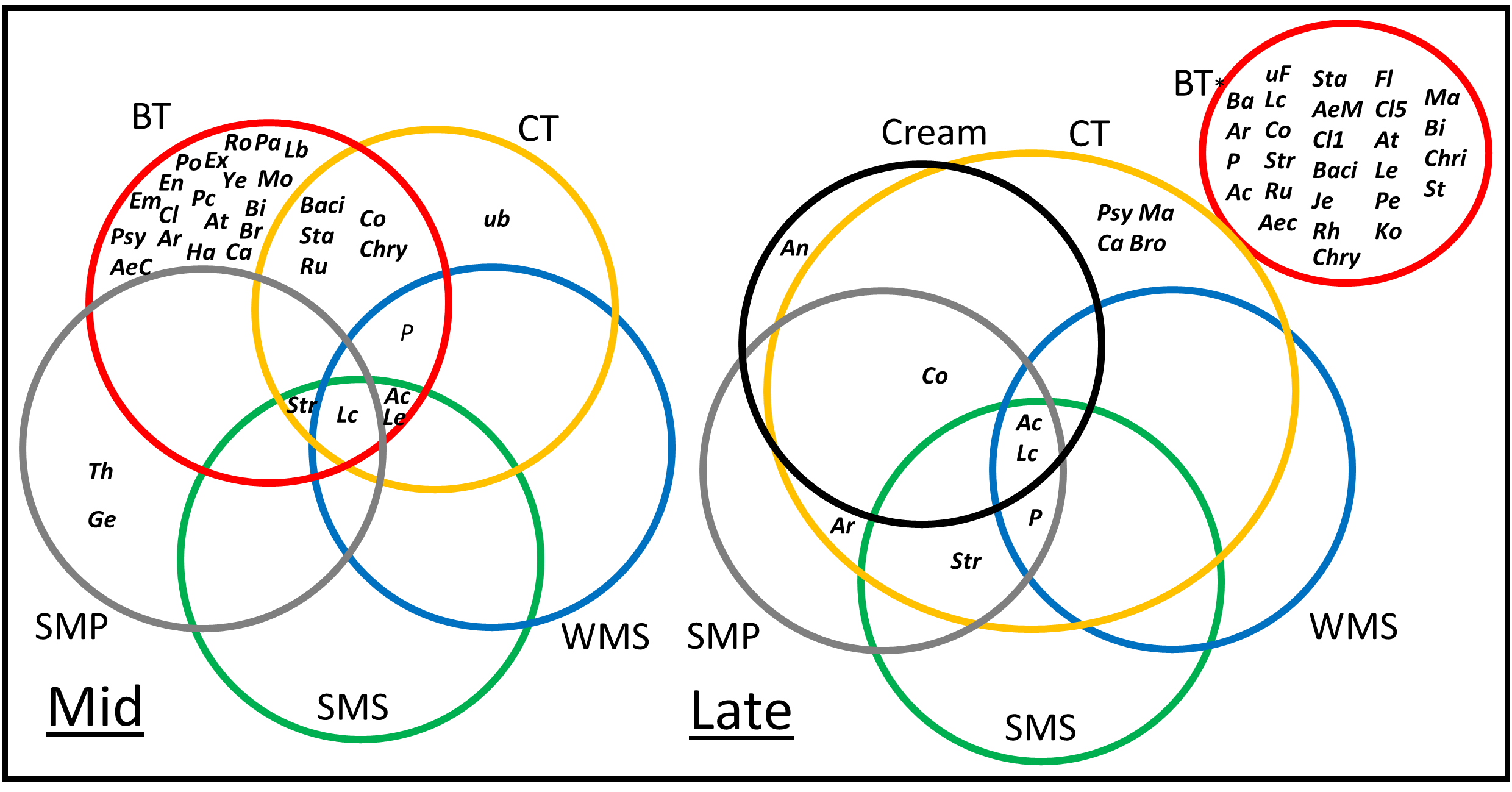

Supplement: FIG S5 [file mSystems.00226-20-sf005.tif]
