# Supplementary material for: Tracking the Dairy Microbiota from Farm Bulk Tank to Skimmed Milk Powder
Source: mSystems. 2020 Apr 7;5(2):e00226-20. doi: 10.1128/mSystems.00226-20 (PMC7141888; doi:10.1128/mSystems.00226-20)
Supplement: TABLE S1 [file mSystems.00226-20-st001.docx]

|  | | **Mid lactation** | |  | |  | **Late lactation** | |  | |  | |
| --- | --- | --- | --- | --- | --- | --- | --- | --- | --- | --- | --- | --- |
| **Genera** | **Stat** | **BT** | **CT** | **WMS** | **SMS** | **SMP** | **BT** | **CT** | **WMS** | **SMS** | **SMP** | **Cream** |
| *Pseudomonas* | Mean (± SD) | ***6.58E-02 (± 9.01E-02)*** | ***7.24E-02 (± 6.29E-02)*** | ***2.73E-01 (± 2.78E-02)*** | 4.07E-02 (± 4.52E-04) | 1.49E-02 (± 9.27E-03) | ***6.59E-02 (± 1.03E-01)*** | ***2.03E-01 (± 9.77E-02)*** | ***2.19E-01 (± 1.23E-02)*** | ***5.49E-01 (± 3.54E-03)*** | ***3.43E-01 (± 2.07E-02)*** | 4.49E-02 |
| *Pseudomonas* | Min ; Max | ***3.41E-03 ; 4.35E-01*** | ***1.56E-02 ; 2.32E-01*** | ***2.53E-01 ; 2.92E-01*** | 4.03E-02 ; 4.10E-02 | 7.34E-03 ; 3.24E-02 | ***2.35E-03 ; 5.19E-01*** | ***1.01E-01 ; 4.19E-01*** | ***2.11E-01 ; 2.28E-01*** | ***5.47E-01 ; 5.52E-01*** | ***3.09E-01 ; 3.86E-01*** | 4.49E-02 |
| *Acinetobacter* | Mean (± SD) | ***6.23E-02 (± 8.42E-02)*** | ***9.65E-02 (± 1.08E-01)*** | ***4.13E-01 (± 1.15E-04)*** | ***7.73E-02 (± 3.99E-03)*** | 2.95E-02 (± 9.13E-03) | ***4.14E-02 (± 9.09E-02)*** | ***2.54E-01 (± 2.17E-01)*** | ***5.31E-01 (± 3.61E-03)*** | ***6.68E-02 (± 1.24E-03)*** | ***2.14E-01 (± 2.62E-02)*** | ***7.69E-02*** |
| *Acinetobacter* | Min ; Max | ***3.19E-03 ; 3.77E-01*** | ***1.36E-02 ; 3.04E-01*** | ***4.13E-01 ; 4.13E-01*** | ***7.44E-02 ; 8.01E-02*** | 1.71E-02 ; 4.80E-02 | ***3.08E-03 ; 6.60E-01*** | ***3.19E-02 ; 7.40E-01*** | ***5.28E-01 ; 5.33E-01*** | ***6.60E-02 ; 6.77E-02*** | ***1.83E-01 ; 2.43E-01*** | ***7.69E-02*** |
| *Lactococcus* | Mean (± SD) | ***5.98E-02 (± 1.05E-01)*** | ***5.21E-02 (± 4.88E-02)*** | ***1.62E-01 (± 9.36E-03)*** | ***1.08E-01 (± 8.24E-03)*** | ***3.05E-02 (± 1.26E-02)*** | ***3.50E-02 (± 4.87E-02)*** | ***7.05E-02 (± 1.23E-01)*** | ***1.05E-01 (± 1.37E-02)*** | ***8.92E-02 (± 9.63E-03)*** | ***5.05E-02 (± 7.02E-03)*** | ***2.27E-01*** |
| *Lactococcus* | Min ; Max | ***2.69E-04 ; 6.19E-01*** | ***5.10E-03 ; 1.36E-01*** | ***1.55E-01 ; 1.68E-01*** | ***1.02E-01 ; 1.14E-01*** | ***1.45E-02 ; 5.01E-02*** | ***5.58E-04 ; 2.62E-01*** | ***2.57E-03 ; 3.93E-01*** | ***9.55E-02 ; 1.15E-01*** | ***8.24E-02 ; 9.60E-02*** | ***3.56E-02 ; 5.72E-02*** | ***2.27E-01*** |
| *Corynebacterium 1* | Mean (± SD) | ***3.30E-02 (± 2.70E-02)*** | ***3.28E-02 (± 1.86E-02)*** | 1.65E-03 (± 4.74E-04) | 4.02E-02 (± 6.95E-04) | 5.05E-03 (± 3.03E-03) | ***5.06E-02 (± 3.09E-02)*** | ***5.59E-02 (± 3.82E-02)*** | 1.68E-02 (± 8.05E-04) | 3.65E-02 (± 6.81E-04) | ***7.85E-02 (± 8.42E-03)*** | ***5.96E-02*** |
| *Corynebacterium 1* | Min ; Max | ***3.94E-04 ; 1.37E-01*** | ***2.98E-03 ; 5.67E-02*** | 1.31E-03 ; 1.99E-03 | 3.97E-02 ; 4.07E-02 | 2.01E-03 ; 1.05E-02 | ***4.44E-03 ; 1.49E-01*** | ***4.97E-03 ; 1.31E-01*** | 1.62E-02 ; 1.73E-02 | 3.60E-02 ; 3.70E-02 | ***6.88E-02 ; 9.36E-02*** | ***5.96E-02*** |
| *Streptococcus* | Mean (± SD) | ***3.49E-02 (± 6.91E-02)*** | ***6.94E-02 (± 7.63E-02)*** | 2.71E-03 (± 1.32E-03) | ***8.53E-02 (± 1.43E-04)*** | ***1.60E-01 (± 5.70E-02)*** | ***1.45E-02 (± 3.15E-02)*** | ***1.95E-02 (± 2.56E-02)*** | 8.61E-03 (± 2.99E-04) | ***5.36E-02 (± 8.05E-03)*** | ***4.60E-02 (± 5.80E-03)*** | 3.33E-02 |
| *Streptococcus* | Min ; Max | ***8.40E-04 ; 3.61E-01*** | ***5.09E-03 ; 2.56E-01*** | 1.78E-03 ; 3.65E-03 | ***8.52E-02 ; 8.54E-02*** | ***6.80E-02 ; 2.13E-01*** | ***8.30E-04 ; 2.51E-01*** | ***8.62E-04 ; 8.63E-02*** | 8.40E-03 ; 8.82E-03 | ***4.79E-02 ; 5.93E-02*** | ***3.82E-02 ; 5.54E-02*** | 3.33E-02 |
| *Ruminococcaceae UCG.005* | Mean (± SD) | ***3.83E-02 (± 2.10E-02)*** | ***3.92E-02 (± 1.42E-02)*** | 1.64E-03 (± 6.45E-04) | 3.31E-02 (± 1.81E-03) | 3.88E-04 (± 1.31E-04) | ***3.78E-02 (± 1.58E-02)*** | 1.48E-02 (± 1.07E-02) | 3.13E-03 (± 1.68E-04) | 4.21E-03 (± 5.13E-04) | 2.19E-03 (± 1.04E-03) | 7.57E-03 |
| *Ruminococcaceae UCG.005* | Min ; Max | ***5.72E-04 ; 8.64E-02*** | ***5.72E-03 ; 5.20E-02*** | 1.19E-03 ; 2.10E-03 | 3.19E-02 ; 3.44E-02 | 2.39E-04 ; 5.73E-04 | ***6.42E-04 ; 7.87E-02*** | 1.09E-03 ; 3.15E-02 | 3.01E-03 ; 3.25E-03 | 3.85E-03 ; 4.57E-03 | 1.42E-03 ; 4.58E-03 | 7.57E-03 |
| *Staphylococcus* | Mean (± SD) | ***3.82E-02 (± 6.78E-02)*** | ***3.14E-02 (± 3.82E-02)*** | 2.06E-03 (± 8.30E-04) | 3.25E-02 (± 4.57E-03) | 2.37E-03 (± 1.01E-03) | ***2.63E-02 (± 3.71E-02)*** | 6.35E-03 (± 8.45E-03) | 4.12E-03 (± 5.86E-04) | 3.63E-03 (± 2.26E-04) | 7.96E-03 (± 2.11E-03) | 1.19E-02 |
| *Staphylococcus* | Min ; Max | ***1.18E-03 ; 4.81E-01*** | ***3.06E-03 ; 1.35E-01*** | 1.47E-03 ; 2.64E-03 | 2.93E-02 ; 3.58E-02 | 1.35E-03 ; 4.21E-03 | ***2.43E-03 ; 2.70E-01*** | 5.24E-04 ; 3.07E-02 | 3.71E-03 ; 4.54E-03 | 3.47E-03 ; 3.79E-03 | 5.07E-03 ; 1.18E-02 | 1.19E-02 |
| *Geobacillus* | Mean (± SD) | 5.19E-04 (± 9.64E-04) | 3.35E-03 (± 4.86E-03) | 1.87E-04 (± 2.52E-04) | 2.06E-03 (± 2.37E-03) | ***4.61E-01 (± 7.12E-02)*** | 2.78E-04 (± 4.12E-04) | 1.04E-04 (± 8.51E-05) | 4.41E-05 (± 2.26E-05) | 5.26E-05 (± 7.44E-05) | 1.38E-04 (± 1.01E-04) | 5.12E-05 |
| *Geobacillus* | Min ; Max | 0.00E+00 ; 5.74E-03 | 1.26E-04 ; 1.44E-02 | 8.59E-06 ; 3.65E-04 | 3.78E-04 ; 3.73E-03 | ***4.04E-01 ; 6.04E-01*** | 0.00E+00 ; 1.86E-03 | 3.02E-05 ; 3.18E-04 | 2.82E-05 ; 6.01E-05 | 0.00E+00 ; 1.05E-04 | 6.04E-05 ; 3.72E-04 | 5.12E-05 |
| *Psychrobacter* | Mean (± SD) | ***4.01E-02 (± 5.15E-02)*** | 1.33E-02 (± 8.77E-03) | 1.07E-03 (± 6.43E-04) | 1.23E-02 (± 4.44E-04) | 1.16E-03 (± 7.34E-04) | 6.01E-03 (± 7.91E-03) | ***2.44E-02 (± 2.23E-02)*** | 4.36E-03 (± 7.58E-05) | 4.17E-03 (± 7.56E-04) | 2.67E-03 (± 4.61E-04) | 4.75E-03 |
| *Psychrobacter* | Min ; Max | ***4.73E-04 ; 2.82E-01*** | 3.80E-03 ; 3.27E-02 | 6.19E-04 ; 1.53E-03 | 1.20E-02 ; 1.26E-02 | 4.13E-04 ; 2.54E-03 | 7.05E-06 ; 4.10E-02 | ***6.77E-04 ; 6.99E-02*** | 4.31E-03 ; 4.42E-03 | 3.64E-03 ; 4.71E-03 | 2.15E-03 ; 3.66E-03 | 4.75E-03 |
| *Arthrobacter* | Mean (± SD) | ***2.01E-02 (± 1.37E-02)*** | 9.50E-03 (± 5.49E-03) | 1.28E-03 (± 2.74E-04) | 6.34E-03 (± 8.55E-04) | 4.27E-04 (± 2.16E-04) | ***1.33E-02 (± 1.08E-02)*** | ***1.82E-02 (± 1.86E-02)*** | 6.07E-03 (± 4.35E-04) | 1.39E-02 (± 1.00E-03) | ***6.53E-02 (± 6.73E-03)*** | 7.71E-03 |
| *Arthrobacter* | Min ; Max | ***9.28E-04 ; 7.02E-02*** | 1.63E-03 ; 1.99E-02 | 1.09E-03 ; 1.48E-03 | 5.73E-03 ; 6.94E-03 | 2.31E-04 ; 8.55E-04 | ***8.70E-04 ; 7.57E-02*** | ***1.08E-03 ; 5.94E-02*** | 5.76E-03 ; 6.38E-03 | 1.32E-02 ; 1.46E-02 | ***5.56E-02 ; 7.40E-02*** | 7.71E-03 |
| *Christensenellaceae R.7 group* | Mean (± SD) | 1.81E-02 (± 1.03E-02) | 1.66E-02 (± 7.17E-03) | 9.70E-04 (± 3.52E-04) | 1.39E-02 (± 1.03E-03) | 3.00E-04 (± 1.22E-04) | ***2.09E-02 (± 1.03E-02)*** | 7.19E-03 (± 4.75E-03) | 2.08E-03 (± 6.56E-05) | 2.51E-03 (± 5.74E-05) | 1.61E-03 (± 6.78E-04) | 6.28E-03 |
| *Christensenellaceae R.7 group* | Min ; Max | 2.80E-04 ; 3.81E-02 | 2.28E-03 ; 2.92E-02 | 7.22E-04 ; 1.22E-03 | 1.32E-02 ; 1.47E-02 | 1.16E-04 ; 4.79E-04 | ***7.85E-04 ; 7.29E-02*** | 5.73E-04 ; 1.34E-02 | 2.04E-03 ; 2.13E-03 | 2.47E-03 ; 2.55E-03 | 9.46E-04 ; 2.92E-03 | 6.28E-03 |
| *Bacteroides* | Mean (± SD) | 1.87E-02 (± 1.08E-02) | 1.74E-02 (± 7.01E-03) | 8.98E-04 (± 2.86E-04) | 1.66E-02 (± 4.52E-03) | 1.48E-03 (± 9.40E-04) | ***1.94E-02 (± 1.46E-02)*** | 7.95E-03 (± 6.38E-03) | 1.43E-03 (± 1.39E-04) | 1.79E-03 (± 3.75E-04) | 1.77E-03 (± 3.45E-04) | 4.20E-03 |
| *Bacteroides* | Min ; Max | 3.43E-04 ; 4.24E-02 | 6.37E-03 ; 2.61E-02 | 6.96E-04 ; 1.10E-03 | 1.34E-02 ; 1.98E-02 | 3.61E-04 ; 2.94E-03 | ***3.57E-04 ; 1.14E-01*** | 5.10E-04 ; 1.77E-02 | 1.33E-03 ; 1.53E-03 | 1.52E-03 ; 2.05E-03 | 1.33E-03 ; 2.51E-03 | 4.20E-03 |
| *Chryseobacterium* | Mean (± SD) | ***2.37E-02 (± 3.85E-02)*** | ***3.72E-02 (± 5.86E-02)*** | 1.52E-02 (± 1.06E-03) | 1.71E-02 (± 1.84E-03) | 9.86E-05 (± 7.64E-05) | ***9.89E-03 (± 1.35E-02)*** | 5.94E-03 (± 7.18E-03) | 1.08E-03 (± 5.01E-04) | 4.15E-04 (± 2.11E-05) | 1.15E-04 (± 6.35E-05) | 5.63E-04 |
| *Chryseobacterium* | Min ; Max | ***8.33E-04 ; 2.63E-01*** | ***3.91E-03 ; 1.63E-01*** | 1.45E-02 ; 1.60E-02 | 1.57E-02 ; 1.84E-02 | 2.48E-05 ; 2.83E-04 | ***4.02E-04 ; 7.45E-02*** | 2.18E-04 ; 2.07E-02 | 7.21E-04 ; 1.43E-03 | 4.00E-04 ; 4.30E-04 | 2.40E-05 ; 2.08E-04 | 5.63E-04 |
| *Leuconostoc* | Mean (± SD) | ***1.80E-02 (± 5.86E-02)*** | ***3.68E-02 (± 7.55E-02)*** | ***6.26E-02 (± 1.11E-02)*** | ***5.10E-02 (± 9.88E-03)*** | 4.32E-03 (± 2.93E-03) | ***6.00E-03 (± 1.64E-02)*** | 4.33E-03 (± 4.99E-03) | 1.06E-03 (± 3.28E-04) | 1.56E-03 (± 5.25E-04) | 7.46E-04 (± 1.90E-04) | 8.53E-04 |
| *Leuconostoc* | Min ; Max | ***1.27E-05 ; 3.95E-01*** | ***2.96E-04 ; 2.49E-01*** | ***5.48E-02 ; 7.05E-02*** | ***4.40E-02 ; 5.80E-02*** | 1.29E-03 ; 9.34E-03 | ***0.00E+00 ; 9.75E-02*** | 1.92E-04 ; 1.84E-02 | 8.33E-04 ; 1.30E-03 | 1.19E-03 ; 1.93E-03 | 4.83E-04 ; 1.07E-03 | 8.53E-04 |
| *Thermus* | Mean (± SD) | 1.84E-05 (± 4.16E-05) | 9.91E-05 (± 1.80E-04) | 5.06E-05 (± 5.94E-05) | 1.78E-04 (± 7.71E-05) | ***2.54E-01 (± 4.69E-02)*** | 1.98E-04 (± 5.68E-04) | 1.86E-05 (± 1.41E-05) | 4.29E-06 (± 6.07E-06) | 0.00E+00 (± 0.00E+00) | 4.21E-05 (± 4.14E-05) | 1.71E-05 |
| *Thermus* | Min ; Max | 0.00E+00 ; 2.17E-04 | 0.00E+00 ; 5.54E-04 | 8.59E-06 ; 9.26E-05 | 1.23E-04 ; 2.32E-04 | ***1.76E-01 ; 3.12E-01*** | 0.00E+00 ; 3.64E-03 | 0.00E+00 ; 3.46E-05 | 0.00E+00 ; 8.59E-06 | 0.00E+00 ; 0.00E+00 | 0.00E+00 ; 1.33E-04 | 1.71E-05 |
| *Peptoclostridium* | Mean (± SD) | 1.23E-02 (± 6.92E-03) | 1.15E-02 (± 4.11E-03) | 7.44E-04 (± 5.18E-04) | 3.46E-02 (± 8.21E-05) | 2.11E-03 (± 7.67E-04) | ***1.49E-02 (± 1.20E-02)*** | 3.63E-03 (± 2.77E-03) | 1.12E-03 (± 7.41E-05) | 7.61E-03 (± 4.57E-04) | 8.37E-03 (± 2.15E-03) | 2.02E-02 |
| *Peptoclostridium* | Min ; Max | 2.54E-04 ; 3.16E-02 | 1.87E-03 ; 1.58E-02 | 3.78E-04 ; 1.11E-03 | 3.46E-02 ; 3.47E-02 | 1.39E-03 ; 3.82E-03 | ***1.43E-04 ; 6.94E-02*** | 3.26E-04 ; 8.04E-03 | 1.06E-03 ; 1.17E-03 | 7.29E-03 ; 7.94E-03 | 5.97E-03 ; 1.25E-02 | 2.02E-02 |
| *Kocuria* | Mean (± SD) | 7.43E-03 (± 7.95E-03) | 1.29E-02 (± 1.56E-02) | 3.30E-04 (± 7.84E-05) | 1.61E-02 (± 4.05E-04) | 1.21E-03 (± 6.98E-04) | ***8.56E-03 (± 8.75E-03)*** | 3.82E-03 (± 2.20E-03) | 1.44E-03 (± 3.42E-04) | 2.53E-03 (± 2.35E-05) | 6.03E-03 (± 5.36E-04) | 4.92E-03 |
| *Kocuria* | Min ; Max | 5.08E-05 ; 3.12E-02 | 4.45E-04 ; 4.36E-02 | 2.75E-04 ; 3.86E-04 | 1.58E-02 ; 1.64E-02 | 4.13E-04 ; 2.25E-03 | ***4.70E-04 ; 5.58E-02*** | 3.08E-04 ; 6.37E-03 | 1.19E-03 ; 1.68E-03 | 2.51E-03 ; 2.54E-03 | 5.29E-03 ; 6.81E-03 | 4.92E-03 |
| *Aerococcus* | Mean (± SD) | ***6.18E-03 (± 1.33E-02)*** | 4.49E-03 (± 2.36E-03) | 6.53E-04 (± 3.62E-05) | 5.59E-03 (± 2.84E-04) | 4.33E-04 (± 3.05E-04) | ***9.35E-03 (± 9.57E-03)*** | 8.52E-03 (± 5.99E-03) | 3.81E-03 (± 7.94E-04) | 6.83E-03 (± 1.01E-04) | 9.40E-03 (± 1.68E-03) | 7.23E-03 |
| *Aerococcus* | Min ; Max | ***1.94E-04 ; 1.08E-01*** | 1.55E-03 ; 8.74E-03 | 6.27E-04 ; 6.79E-04 | 5.39E-03 ; 5.79E-03 | 1.62E-04 ; 1.02E-03 | ***1.57E-04 ; 6.42E-02*** | 6.00E-04 ; 2.22E-02 | 3.25E-03 ; 4.37E-03 | 6.76E-03 ; 6.90E-03 | 7.11E-03 ; 1.17E-02 | 7.23E-03 |
| *Clostridium sensu stricto 1* | Mean (± SD) | 4.56E-03 (± 3.30E-03) | 4.47E-03 (± 2.16E-03) | 1.96E-04 (± 1.08E-04) | 9.02E-03 (± 1.63E-03) | 5.68E-04 (± 2.60E-04) | ***1.21E-02 (± 1.88E-02)*** | 1.67E-03 (± 1.32E-03) | 3.70E-04 (± 7.43E-05) | 1.69E-03 (± 1.46E-04) | 1.68E-03 (± 6.40E-04) | 5.56E-03 |
| *Clostridium sensu stricto 1* | Min ; Max | 7.62E-05 ; 1.61E-02 | 1.20E-03 ; 8.90E-03 | 1.20E-04 ; 2.73E-04 | 7.87E-03 ; 1.02E-02 | 2.80E-04 ; 9.73E-04 | ***3.14E-04 ; 9.77E-02*** | 1.49E-04 ; 4.13E-03 | 3.18E-04 ; 4.23E-04 | 1.59E-03 ; 1.79E-03 | 1.21E-03 ; 3.20E-03 | 5.56E-03 |
| *Bacillus* | Mean (± SD) | ***6.46E-03 (± 9.48E-03)*** | ***1.23E-02 (± 1.75E-02)*** | 4.66E-04 (± 5.99E-04) | 6.39E-03 (± 6.00E-03) | 4.49E-04 (± 2.41E-04) | ***8.49E-03 (± 1.03E-02)*** | 6.68E-04 (± 4.15E-04) | 4.18E-04 (± 2.22E-04) | 4.78E-04 (± 2.18E-04) | 5.98E-04 (± 3.39E-04) | 2.06E-02 |
| *Bacillus* | Min ; Max | ***5.08E-05 ; 5.81E-02*** | ***1.20E-03 ; 5.62E-02*** | 4.30E-05 ; 8.90E-04 | 2.15E-03 ; 1.06E-02 | 2.31E-04 ; 9.35E-04 | ***3.57E-04 ; 5.90E-02*** | 1.46E-04 ; 1.19E-03 | 2.61E-04 ; 5.75E-04 | 3.25E-04 ; 6.32E-04 | 3.24E-04 ; 1.45E-03 | 2.06E-02 |
| *Jeotgalicoccus* | Mean (± SD) | 4.26E-03 (± 4.07E-03) | 2.95E-03 (± 2.04E-03) | 1.39E-04 (± 8.72E-05) | 3.25E-03 (± 2.86E-04) | 3.58E-05 (± 2.42E-05) | ***9.65E-03 (± 1.21E-02)*** | 1.11E-02 (± 9.96E-03) | 2.74E-03 (± 3.70E-04) | 4.12E-03 (± 3.00E-04) | 2.67E-03 (± 4.36E-04) | 4.78E-03 |
| *Jeotgalicoccus* | Min ; Max | 3.81E-05 ; 2.14E-02 | 2.00E-04 ; 6.33E-03 | 7.73E-05 ; 2.01E-04 | 3.05E-03 ; 3.45E-03 | 0.00E+00 ; 7.43E-05 | ***2.28E-04 ; 8.10E-02*** | 8.99E-04 ; 3.57E-02 | 2.47E-03 ; 3.00E-03 | 3.91E-03 ; 4.33E-03 | 2.15E-03 ; 3.46E-03 | 4.78E-03 |
| *Lactobacillus* | Mean (± SD) | ***5.05E-03 (± 8.99E-03)*** | 2.02E-03 (± 3.15E-03) | 4.88E-04 (± 4.59E-04) | 7.37E-03 (± 2.62E-03) | 1.67E-03 (± 8.29E-04) | 5.41E-03 (± 7.65E-03) | 1.47E-03 (± 1.11E-03) | 9.15E-04 (± 4.20E-04) | 4.16E-03 (± 8.80E-04) | 1.35E-02 (± 4.16E-03) | 3.80E-03 |
| *Lactobacillus* | Min ; Max | ***0.00E+00 ; 6.04E-02*** | 3.09E-04 ; 1.13E-02 | 1.63E-04 ; 8.13E-04 | 5.52E-03 ; 9.23E-03 | 8.54E-04 ; 3.16E-03 | 1.39E-04 ; 4.58E-02 | 1.39E-04 ; 4.15E-03 | 6.18E-04 ; 1.21E-03 | 3.54E-03 ; 4.78E-03 | 9.60E-03 ; 2.18E-02 | 3.80E-03 |
| *Paracocccus* | Mean (± SD) | ***7.48E-03 (± 1.20E-02)*** | 2.12E-03 (± 2.03E-03) | 2.18E-04 (± 1.87E-04) | 3.63E-04 (± 2.46E-04) | 2.97E-05 (± 2.76E-05) | 4.68E-03 (± 2.74E-03) | 1.06E-03 (± 1.09E-03) | 1.88E-04 (± 7.22E-05) | 2.71E-04 (± 1.10E-04) | 1.90E-04 (± 7.67E-05) | 1.06E-03 |
| *Paracocccus* | Min ; Max | ***6.35E-05 ; 7.91E-02*** | 8.54E-05 ; 6.36E-03 | 8.59E-05 ; 3.50E-04 | 1.89E-04 ; 5.37E-04 | 0.00E+00 ; 8.63E-05 | 2.28E-04 ; 1.26E-02 | 0.00E+00 ; 2.66E-03 | 1.37E-04 ; 2.40E-04 | 1.93E-04 ; 3.48E-04 | 6.94E-05 ; 3.24E-04 | 1.06E-03 |
| *Rhodococcus* | Mean (± SD) | 6.25E-03 (± 6.89E-03) | 3.36E-03 (± 2.63E-03) | 3.04E-04 (± 8.06E-05) | 2.74E-03 (± 1.13E-03) | 2.89E-04 (± 1.74E-04) | ***4.88E-03 (± 8.04E-03)*** | 2.11E-03 (± 2.72E-03) | 6.45E-04 (± 1.40E-05) | 1.54E-03 (± 1.05E-04) | 2.73E-03 (± 2.58E-04) | 1.80E-03 |
| *Rhodococcus* | Min ; Max | 2.54E-05 ; 3.33E-02 | 4.76E-04 ; 8.25E-03 | 2.47E-04 ; 3.61E-04 | 1.95E-03 ; 3.54E-03 | 1.16E-04 ; 5.89E-04 | ***2.95E-04 ; 6.53E-02*** | 2.46E-04 ; 9.25E-03 | 6.35E-04 ; 6.55E-04 | 1.46E-03 ; 1.61E-03 | 2.46E-03 ; 3.36E-03 | 1.80E-03 |
| *Moraxella* | Mean (± SD) | ***7.18E-03 (± 1.45E-02)*** | 4.32E-03 (± 4.99E-03) | 3.17E-04 (± 8.32E-05) | 2.04E-02 (± 2.17E-03) | 1.74E-03 (± 1.13E-03) | 2.85E-03 (± 4.24E-03) | 2.07E-03 (± 3.66E-03) | 1.09E-03 (± 4.99E-04) | 4.65E-04 (± 3.71E-05) | 7.52E-04 (± 2.25E-04) | 3.92E-04 |
| *Moraxella* | Min ; Max | ***4.77E-05 ; 8.79E-02*** | 1.13E-04 ; 1.59E-02 | 2.58E-04 ; 3.75E-04 | 1.88E-02 ; 2.19E-02 | 5.20E-04 ; 3.96E-03 | 2.77E-05 ; 2.74E-02 | 0.00E+00 ; 1.02E-02 | 7.38E-04 ; 1.44E-03 | 4.39E-04 ; 4.91E-04 | 5.43E-04 ; 1.26E-03 | 3.92E-04 |
| *Macrococcus* | Mean (± SD) | 2.61E-03 (± 3.87E-03) | 1.37E-03 (± 1.02E-03) | 1.89E-04 (± 1.24E-05) | 2.35E-03 (± 5.38E-05) | 1.56E-04 (± 9.78E-05) | ***7.32E-03 (± 1.05E-02)*** | ***8.01E-03 (± 2.35E-02)*** | 1.84E-03 (± 5.86E-04) | 1.31E-03 (± 1.97E-04) | 2.08E-03 (± 7.02E-04) | 8.70E-04 |
| *Macrococcus* | Min ; Max | 1.23E-04 ; 2.46E-02 | 3.55E-04 ; 3.29E-03 | 1.80E-04 ; 1.98E-04 | 2.31E-03 ; 2.38E-03 | 4.42E-05 ; 3.30E-04 | ***8.04E-06 ; 7.08E-02*** | ***1.94E-04 ; 7.88E-02*** | 1.43E-03 ; 2.25E-03 | 1.17E-03 ; 1.45E-03 | 1.10E-03 ; 3.49E-03 | 8.70E-04 |
| *Rothia* | Mean (± SD) | ***6.49E-03 (± 1.64E-02)*** | 6.50E-03 (± 8.39E-03) | 5.52E-04 (± 3.68E-04) | 7.25E-03 (± 1.27E-03) | 8.92E-04 (± 2.11E-04) | 2.32E-03 (± 4.38E-03) | 2.49E-03 (± 4.64E-03) | 6.33E-04 (± 5.30E-04) | 4.56E-04 (± 6.16E-05) | 4.72E-04 (± 9.73E-05) | 5.54E-04 |
| *Rothia* | Min ; Max | ***1.72E-05 ; 9.68E-02*** | 3.35E-04 ; 2.48E-02 | 2.92E-04 ; 8.13E-04 | 6.36E-03 ; 8.15E-03 | 5.68E-04 ; 1.35E-03 | 1.61E-05 ; 3.47E-02 | 8.83E-05 ; 1.50E-02 | 2.58E-04 ; 1.01E-03 | 4.13E-04 ; 5.00E-04 | 3.40E-04 ; 6.37E-04 | 5.54E-04 |
| *Bifidobacterium* | Mean (± SD) | ***4.38E-03 (± 1.01E-02)*** | 4.55E-03 (± 6.04E-03) | 2.89E-04 (± 2.39E-04) | 1.02E-02 (± 1.92E-03) | 1.07E-03 (± 7.45E-04) | ***3.54E-03 (± 1.20E-02)*** | 5.71E-03 (± 6.21E-03) | 1.36E-03 (± 2.77E-04) | 2.06E-03 (± 2.71E-04) | 2.07E-03 (± 2.78E-04) | 1.73E-03 |
| *Bifidobacterium* | Min ; Max | ***0.00E+00 ; 5.86E-02*** | 2.30E-04 ; 2.24E-02 | 1.20E-04 ; 4.58E-04 | 8.89E-03 ; 1.16E-02 | 3.97E-04 ; 2.55E-03 | ***0.00E+00 ; 8.57E-02*** | 1.23E-04 ; 1.86E-02 | 1.16E-03 ; 1.55E-03 | 1.87E-03 ; 2.25E-03 | 1.70E-03 ; 2.39E-03 | 1.73E-03 |
| *Flavobacterium* | Mean (± SD) | 4.46E-03 (± 7.18E-03) | 2.29E-03 (± 2.55E-03) | 5.61E-04 (± 6.11E-04) | 2.45E-03 (± 1.08E-03) | 1.69E-04 (± 9.03E-05) | ***3.87E-03 (± 7.93E-03)*** | 5.08E-03 (± 7.39E-03) | 2.44E-03 (± 6.24E-04) | 7.92E-04 (± 1.40E-04) | 2.35E-04 (± 9.40E-05) | 7.51E-04 |
| *Flavobacterium* | Min ; Max | 0.00E+00 ; 3.79E-02 | 1.42E-04 ; 8.46E-03 | 1.29E-04 ; 9.93E-04 | 1.68E-03 ; 3.21E-03 | 7.16E-05 ; 3.61E-04 | ***8.56E-05 ; 6.66E-02*** | 1.72E-04 ; 2.53E-02 | 1.99E-03 ; 2.88E-03 | 6.93E-04 ; 8.90E-04 | 6.43E-05 ; 3.75E-04 | 7.51E-04 |
| *Brachybacterium* | Mean (± SD) | ***5.35E-03 (± 1.16E-02)*** | 2.78E-03 (± 3.73E-03) | 9.78E-05 (± 6.54E-05) | 1.55E-03 (± 2.35E-04) | 1.69E-04 (± 9.42E-05) | 3.39E-03 (± 4.81E-03) | 1.53E-03 (± 1.24E-03) | 6.04E-04 (± 1.02E-04) | 5.87E-04 (± 1.37E-04) | 1.06E-03 (± 2.74E-04) | 1.82E-03 |
| *Brachybacterium* | Min ; Max | ***6.35E-05 ; 8.75E-02*** | 2.76E-04 ; 1.18E-02 | 5.15E-05 ; 1.44E-04 | 1.38E-03 ; 1.71E-03 | 3.98E-05 ; 3.16E-04 | 1.97E-04 ; 2.59E-02 | 9.24E-05 ; 3.45E-03 | 5.32E-04 ; 6.76E-04 | 4.90E-04 ; 6.84E-04 | 6.73E-04 ; 1.61E-03 | 1.82E-03 |
| *Enterococcus* | Mean (± SD) | ***4.19E-03 (± 9.85E-03)*** | 5.29E-03 (± 7.99E-03) | 1.95E-04 (± 1.54E-04) | 3.40E-03 (± 7.75E-04) | 5.34E-04 (± 2.83E-04) | 2.42E-03 (± 3.86E-03) | 1.31E-03 (± 1.13E-03) | 5.32E-04 (± 8.46E-05) | 1.57E-03 (± 6.47E-05) | 3.00E-03 (± 7.11E-04) | 8.44E-04 |
| *Enterococcus* | Min ; Max | ***1.08E-04 ; 6.16E-02*** | 7.37E-04 ; 2.86E-02 | 8.59E-05 ; 3.03E-04 | 2.85E-03 ; 3.95E-03 | 2.74E-04 ; 1.22E-03 | 0.00E+00 ; 2.39E-02 | 6.16E-05 ; 3.84E-03 | 4.72E-04 ; 5.92E-04 | 1.52E-03 ; 1.61E-03 | 1.70E-03 ; 3.89E-03 | 8.44E-04 |
| *Pseudoclavibacter* | Mean (± SD) | ***5.73E-03 (± 1.24E-02)*** | 4.72E-03 (± 9.91E-03) | 1.11E-04 (± 1.07E-05) | 7.73E-04 (± 4.77E-04) | 6.13E-05 (± 4.38E-05) | 1.01E-03 (± 1.22E-03) | 1.01E-03 (± 1.88E-03) | 2.74E-04 (± 6.03E-05) | 4.31E-04 (± 1.35E-04) | 4.77E-04 (± 1.57E-04) | 2.47E-04 |
| *Pseudoclavibacter* | Min ; Max | ***1.27E-05 ; 6.88E-02*** | 3.94E-05 ; 3.19E-02 | 1.03E-04 ; 1.18E-04 | 4.36E-04 ; 1.11E-03 | 7.36E-06 ; 1.29E-04 | 1.22E-04 ; 6.73E-03 | 2.86E-05 ; 6.56E-03 | 2.32E-04 ; 3.17E-04 | 3.35E-04 ; 5.26E-04 | 2.54E-04 ; 7.21E-04 | 2.47E-04 |
| *Clostridium sensu stricto 5* | Mean (± SD) | 1.45E-05 (± 4.79E-05) | 1.14E-06 (± 2.63E-06) | 0.00E+00 (± 0.00E+00) | 3.00E-05 (± 1.92E-05) | 1.64E-06 (± 4.91E-06) | ***7.41E-03 (± 1.67E-02)*** | 2.79E-05 (± 3.37E-05) | 0.00E+00 (± 0.00E+00) | 0.00E+00 (± 0.00E+00) | 7.11E-05 (± 1.23E-04) | 1.11E-04 |
| *Clostridium sensu stricto 5* | Min ; Max | 0.00E+00 ; 3.47E-04 | 0.00E+00 ; 7.80E-06 | 0.00E+00 ; 0.00E+00 | 1.64E-05 ; 4.36E-05 | 0.00E+00 ; 1.47E-05 | ***0.00E+00 ; 9.54E-02*** | 0.00E+00 ; 1.07E-04 | 0.00E+00 ; 0.00E+00 | 0.00E+00 ; 0.00E+00 | 0.00E+00 ; 3.91E-04 | 1.11E-04 |
| *Clavibacter* | Mean (± SD) | ***5.45E-03 (± 1.08E-02)*** | 4.09E-03 (± 8.70E-03) | 1.66E-04 (± 1.14E-04) | 5.79E-04 (± 1.00E-04) | 9.09E-05 (± 5.96E-05) | 9.68E-04 (± 1.09E-03) | 8.05E-04 (± 1.73E-03) | 1.88E-04 (± 1.33E-04) | 3.40E-04 (± 1.15E-04) | 4.18E-04 (± 2.00E-04) | 2.05E-04 |
| *Clavibacter* | Min ; Max | ***4.39E-05 ; 5.51E-02*** | 2.30E-04 ; 2.96E-02 | 8.59E-05 ; 2.47E-04 | 5.08E-04 ; 6.50E-04 | 3.18E-05 ; 2.12E-04 | 0.00E+00 ; 7.16E-03 | 4.01E-05 ; 5.97E-03 | 9.45E-05 ; 2.82E-04 | 2.58E-04 ; 4.21E-04 | 2.12E-04 ; 8.65E-04 | 2.05E-04 |
| *Carnobacterium* | Mean (± SD) | ***2.62E-03 (± 1.10E-02)*** | 2.41E-03 (± 3.78E-03) | 3.74E-04 (± 2.24E-04) | 2.23E-03 (± 1.14E-03) | 2.37E-04 (± 8.65E-05) | 3.00E-04 (± 6.73E-04) | ***1.37E-02 (± 2.38E-02)*** | 8.65E-03 (± 1.88E-03) | 1.18E-02 (± 2.86E-04) | 4.57E-03 (± 1.22E-03) | 1.14E-02 |
| *Carnobacterium* | Min ; Max | ***0.00E+00 ; 8.65E-02*** | 4.82E-05 ; 1.17E-02 | 2.16E-04 ; 5.33E-04 | 1.42E-03 ; 3.03E-03 | 1.27E-04 ; 3.61E-04 | 0.00E+00 ; 3.42E-03 | ***1.62E-04 ; 7.50E-02*** | 7.32E-03 ; 9.98E-03 | 1.16E-02 ; 1.20E-02 | 2.77E-03 ; 6.37E-03 | 1.14E-02 |
| *Atopococcus* | Mean (± SD) | ***2.11E-03 (± 6.92E-03)*** | 1.23E-03 (± 1.81E-03) | 7.80E-05 (± 8.60E-05) | 1.61E-03 (± 3.77E-04) | 1.27E-04 (± 5.17E-05) | ***3.35E-03 (± 2.01E-02)*** | 1.44E-03 (± 1.81E-03) | 8.65E-04 (± 2.75E-05) | 1.42E-03 (± 1.12E-04) | 1.38E-03 (± 3.53E-04) | 1.59E-03 |
| *Atopococcus* | Min ; Max | ***0.00E+00 ; 5.55E-02*** | 0.00E+00 ; 6.19E-03 | 1.72E-05 ; 1.39E-04 | 1.34E-03 ; 1.87E-03 | 4.05E-05 ; 2.12E-04 | ***0.00E+00 ; 1.65E-01*** | 1.44E-04 ; 5.32E-03 | 8.46E-04 ; 8.84E-04 | 1.34E-03 ; 1.50E-03 | 8.85E-04 ; 1.84E-03 | 1.59E-03 |
| *Aeromonas* | Mean (± SD) | 1.99E-03 (± 5.51E-03) | 1.86E-03 (± 3.68E-03) | 7.70E-04 (± 2.96E-04) | 6.93E-04 (± 2.93E-04) | 4.84E-04 (± 2.01E-04) | ***1.76E-03 (± 7.84E-03)*** | 6.37E-04 (± 8.90E-04) | 2.59E-04 (± 1.72E-04) | 9.44E-04 (± 3.30E-05) | 5.64E-03 (± 6.30E-04) | 5.97E-04 |
| *Aeromonas* | Min ; Max | 0.00E+00 ; 4.30E-02 | 4.60E-05 ; 1.26E-02 | 5.61E-04 ; 9.79E-04 | 4.85E-04 ; 9.00E-04 | 2.41E-04 ; 8.11E-04 | ***0.00E+00 ; 6.28E-02*** | 0.00E+00 ; 2.49E-03 | 1.37E-04 ; 3.80E-04 | 9.21E-04 ; 9.68E-04 | 4.73E-03 ; 6.80E-03 | 5.97E-04 |
| *uncultured Fusobacterium sp.* | Mean (± SD) | 8.81E-04 (± 2.40E-03) | 1.63E-03 (± 1.95E-03) | 1.30E-04 (± 1.31E-05) | 1.06E-02 (± 1.24E-03) | 6.22E-04 (± 4.90E-04) | ***2.61E-03 (± 1.14E-02)*** | 4.11E-03 (± 1.28E-02) | 1.04E-03 (± 3.80E-04) | 1.57E-03 (± 1.45E-04) | 6.58E-04 (± 2.00E-04) | 2.44E-03 |
| *uncultured Fusobacterium sp.* | Min ; Max | 0.00E+00 ; 1.56E-02 | 7.22E-05 ; 6.52E-03 | 1.20E-04 ; 1.39E-04 | 9.68E-03 ; 1.14E-02 | 1.57E-04 ; 1.66E-03 | ***0.00E+00 ; 6.97E-02*** | 0.00E+00 ; 4.27E-02 | 7.68E-04 ; 1.31E-03 | 1.47E-03 ; 1.68E-03 | 3.70E-04 ; 9.49E-04 | 2.44E-03 |
| *uncultured bacterium* | Mean (± SD) | 6.61E-04 (± 3.62E-03) | ***1.17E-02 (± 3.27E-02)*** | 9.50E-04 (± 4.16E-05) | 2.25E-02 (± 2.46E-03) | 1.15E-03 (± 6.97E-04) | 1.12E-03 (± 5.78E-03) | 3.36E-04 (± 9.59E-04) | 4.46E-05 (± 2.66E-05) | 1.33E-03 (± 1.01E-04) | 3.95E-04 (± 1.36E-04) | 2.75E-03 |
| *uncultured bacterium* | Min ; Max | 0.00E+00 ; 2.89E-02 | ***0.00E+00 ; 1.09E-01*** | 9.21E-04 ; 9.79E-04 | 2.08E-02 ; 2.43E-02 | 3.26E-04 ; 2.49E-03 | 0.00E+00 ; 3.46E-02 | 0.00E+00 ; 3.21E-03 | 2.58E-05 ; 6.34E-05 | 1.26E-03 ; 1.41E-03 | 2.31E-04 ; 5.77E-04 | 2.75E-03 |
| *Anoxybacillus* | Mean (± SD) | 3.79E-04 (± 9.22E-04) | 4.78E-05 (± 5.14E-05) | 7.71E-06 (± 1.09E-05) | 2.78E-03 (± 7.04E-04) | 3.08E-03 (± 9.18E-04) | 2.22E-04 (± 4.53E-04) | 2.35E-05 (± 3.64E-05) | 4.29E-06 (± 6.07E-06) | 4.54E-05 (± 2.70E-05) | 9.02E-05 (± 5.41E-05) | ***2.36E-01*** |
| *Anoxybacillus* | Min ; Max | 0.00E+00 ; 5.62E-03 | 0.00E+00 ; 1.69E-04 | 0.00E+00 ; 1.54E-05 | 2.29E-03 ; 3.28E-03 | 1.73E-03 ; 4.51E-03 | 0.00E+00 ; 2.61E-03 | 0.00E+00 ; 1.06E-04 | 0.00E+00 ; 8.59E-06 | 2.63E-05 ; 6.45E-05 | 1.79E-05 ; 1.94E-04 | ***2.36E-01*** |
| *Empedobacter* | Mean (± SD) | ***2.16E-03 (± 7.65E-03)*** | 4.28E-04 (± 9.11E-04) | 2.96E-03 (± 4.12E-03) | 6.44E-04 (± 8.94E-05) | 2.08E-06 (± 4.28E-06) | 1.21E-03 (± 3.07E-03) | 3.58E-03 (± 1.04E-02) | 7.51E-03 (± 2.01E-03) | 2.55E-03 (± 7.39E-04) | 1.31E-04 (± 7.37E-05) | 4.95E-04 |
| *Empedobacter* | Min ; Max | ***0.00E+00 ; 6.08E-02*** | 0.00E+00 ; 3.09E-03 | 4.30E-05 ; 5.87E-03 | 5.81E-04 ; 7.07E-04 | 0.00E+00 ; 1.16E-05 | 0.00E+00 ; 2.15E-02 | 0.00E+00 ; 3.50E-02 | 6.09E-03 ; 8.93E-03 | 2.03E-03 ; 3.07E-03 | 4.59E-05 ; 2.69E-04 | 4.95E-04 |
| *Stenotrophomonas* | Mean (± SD) | 1.38E-03 (± 2.87E-03) | 1.37E-03 (± 1.44E-03) | 3.06E-04 (± 3.60E-04) | 6.33E-04 (± 5.22E-04) | 6.74E-05 (± 6.59E-05) | ***2.65E-03 (± 7.96E-03)*** | 6.36E-04 (± 8.88E-04) | 1.66E-04 (± 1.74E-04) | 7.15E-05 (± 2.67E-05) | 6.68E-05 (± 5.87E-05) | 9.38E-05 |
| *Stenotrophomonas* | Min ; Max | 0.00E+00 ; 1.90E-02 | 6.57E-05 ; 4.18E-03 | 5.15E-05 ; 5.61E-04 | 2.63E-04 ; 1.00E-03 | 0.00E+00 ; 2.04E-04 | ***0.00E+00 ; 6.48E-02*** | 0.00E+00 ; 2.81E-03 | 4.29E-05 ; 2.89E-04 | 5.26E-05 ; 9.03E-05 | 0.00E+00 ; 1.35E-04 | 9.38E-05 |
| *Exiguobacterium* | Mean (± SD) | ***1.80E-03 (± 1.07E-02)*** | 2.59E-03 (± 7.02E-03) | 1.65E-02 (± 1.90E-03) | 1.65E-02 (± 2.33E-03) | 4.82E-04 (± 1.84E-04) | 1.86E-04 (± 3.78E-04) | 5.67E-04 (± 1.84E-03) | 5.39E-05 (± 3.43E-06) | 3.69E-05 (± 2.56E-06) | 1.52E-05 (± 2.01E-05) | 1.71E-05 |
| *Exiguobacterium* | Min ; Max | ***0.00E+00 ; 8.63E-02*** | 0.00E+00 ; 2.33E-02 | 1.52E-02 ; 1.79E-02 | 1.49E-02 ; 1.82E-02 | 2.58E-04 ; 7.85E-04 | 0.00E+00 ; 2.59E-03 | 0.00E+00 ; 6.12E-03 | 5.15E-05 ; 5.64E-05 | 3.51E-05 ; 3.87E-05 | 0.00E+00 ; 5.83E-05 | 1.71E-05 |
| *Porphyromonas* | Mean (± SD) | ***1.40E-03 (± 7.46E-03)*** | 1.17E-03 (± 1.36E-03) | 1.36E-04 (± 2.64E-06) | 4.22E-03 (± 4.14E-04) | 2.46E-04 (± 1.44E-04) | 1.65E-03 (± 5.66E-03) | 4.42E-04 (± 5.40E-04) | 6.53E-05 (± 7.32E-06) | 1.94E-04 (± 2.00E-04) | 7.49E-05 (± 3.32E-05) | 1.88E-04 |
| *Porphyromonas* | Min ; Max | ***0.00E+00 ; 6.13E-02*** | 1.86E-04 ; 4.65E-03 | 1.34E-04 ; 1.37E-04 | 3.93E-03 ; 4.52E-03 | 8.75E-05 ; 4.61E-04 | 0.00E+00 ; 4.39E-02 | 1.15E-05 ; 1.48E-03 | 6.01E-05 ; 7.05E-05 | 5.26E-05 ; 3.35E-04 | 2.50E-05 ; 1.17E-04 | 1.88E-04 |
| *Yersinia* | Mean (± SD) | ***1.52E-03 (± 7.13E-03)*** | 5.59E-04 (± 8.49E-04) | 2.03E-03 (± 2.66E-03) | 6.02E-04 (± 5.03E-04) | 4.43E-04 (± 2.77E-04) | 2.93E-04 (± 1.63E-03) | 2.29E-03 (± 4.51E-03) | 1.46E-03 (± 8.69E-04) | 1.05E-03 (± 3.36E-04) | 2.92E-03 (± 4.42E-04) | 7.16E-04 |
| *Yersinia* | Min ; Max | ***0.00E+00 ; 5.63E-02*** | 0.00E+00 ; 2.82E-03 | 1.49E-04 ; 3.91E-03 | 2.47E-04 ; 9.58E-04 | 1.62E-04 ; 1.04E-03 | 0.00E+00 ; 1.31E-02 | 1.38E-05 ; 1.49E-02 | 8.50E-04 ; 2.08E-03 | 8.16E-04 ; 1.29E-03 | 2.31E-03 ; 3.75E-03 | 7.16E-04 |
| *Brochothrix* | Mean (± SD) | 1.79E-05 (± 1.06E-04) | 4.95E-05 (± 9.22E-05) | 2.92E-05 (± 1.95E-05) | 0.00E+00 (± 0.00E+00) | 0.00E+00 (± 0.00E+00) | 1.75E-06 (± 6.23E-06) | ***1.49E-02 (± 4.71E-02)*** | 1.38E-03 (± 4.54E-04) | 1.57E-03 (± 1.35E-04) | 5.50E-04 (± 2.89E-04) | 2.56E-04 |
| *Brochothrix* | Min ; Max | 0.00E+00 ; 8.51E-04 | 0.00E+00 ; 2.73E-04 | 1.54E-05 ; 4.30E-05 | 0.00E+00 ; 0.00E+00 | 0.00E+00 ; 0.00E+00 | 0.00E+00 ; 2.98E-05 | ***0.00E+00 ; 1.57E-01*** | 1.06E-03 ; 1.70E-03 | 1.47E-03 ; 1.66E-03 | 1.65E-04 ; 9.74E-04 | 2.56E-04 |
| *Hafnia* | Mean (± SD) | ***1.91E-03 (± 1.25E-02)*** | 1.79E-03 (± 5.43E-03) | 9.82E-04 (± 3.49E-04) | 8.31E-04 (± 2.21E-04) | 2.30E-04 (± 1.26E-04) | 1.33E-04 (± 6.61E-04) | 8.86E-05 (± 1.77E-04) | 4.92E-05 (± 3.97E-05) | 9.96E-05 (± 4.16E-05) | 3.67E-04 (± 9.90E-05) | 0.00E+00 |
| *Hafnia* | Min ; Max | ***0.00E+00 ; 1.01E-01*** | 0.00E+00 ; 1.81E-02 | 7.35E-04 ; 1.23E-03 | 6.74E-04 ; 9.87E-04 | 8.10E-05 ; 4.32E-04 | 0.00E+00 ; 3.91E-03 | 0.00E+00 ; 5.90E-04 | 2.11E-05 ; 7.73E-05 | 7.02E-05 ; 1.29E-04 | 2.12E-04 ; 5.36E-04 | 0.00E+00 |
| Others | Mean (± SD) | ***4.04E-01 (± 1.57E-01)*** | ***3.53E-01 (± 1.41E-01)*** | 3.05E-02 (± 1.76E-02) | ***2.65E-01 (± 8.25E-04)*** | 1.60E-02 (± 6.73E-03) | ***5.24E-01 (± 1.47E-01)*** | ***2.03E-01 (± 1.32E-01)*** | ***5.41E-02 (± 9.83E-04)*** | ***1.12E-01 (± 2.27E-03)*** | ***1.13E-01 (± 1.61E-02)*** | ***1.80E-01*** |
| Others | Min ; Max | ***9.96E-03 ; 6.90E-01*** | ***6.35E-02 ; 5.14E-01*** | 1.81E-02 ; 4.29E-02 | ***2.65E-01 ; 2.66E-01*** | 9.08E-03 ; 2.98E-02 | ***3.87E-02 ; 7.49E-01*** | ***2.78E-02 ; 4.11E-01*** | ***5.34E-02 ; 5.48E-02*** | ***1.11E-01 ; 1.14E-01*** | ***9.33E-02 ; 1.32E-01*** | ***1.80E-01*** |
